# A ratiometric fluorescent probe for imaging and quantifying anti-apoptotic effects of GSH under temperature stress†
†Electronic supplementary information (ESI) available: Experimental detail procedures, synthetic procedures and characterization details, reaction kinetics and selectivity, and additional data. See DOI: 10.1039/c7sc02888a



**DOI:** 10.1039/c7sc02888a

**Published:** 2017-08-11

**Authors:** Xiaoyue Han, Xinyu Song, Fabiao Yu, Lingxin Chen

**Affiliations:** a Key Laboratory of Coastal Environmental Processes and Ecological Remediation , Research Centre for Coastal Environmental Engineering and Technology , Yantai Institute of Coastal Zone Research , Chinese Academy of Sciences , Yantai 264003 , China . Email: lxchen@yic.ac.cn; b University of Chinese Academy of Sciences , Beijing 100049 , China

## Abstract

Hypothermia and hyperthermia are cell stressed states resulting from environmental temperature changes, which can abnormally decrease intracellular glutathione (GSH) concentrations and induce apoptosis. As the most abundant intracellular non-protein biothiol, GSH can protect cells from apoptosis. Considering the important roles of GSH in the anti-apoptotic process in cells and *in vivo*, we strive to develop a powerful chemical tool for the direct detection of GSH concentration changes under temperature stress. Herein, we report a ratiometric fluorescent probe (**CyO-Dise**) based on a selenium–sulfur exchange reaction for the qualitative and quantitative detection of GSH concentration fluctuations in cells and *in vivo*. The probe has been successfully used to assess the changes of GSH levels in HepG2 and HL-7702 cells using the stimulations of hypothermia and hyperthermia. In terms of the anti-apoptotic effect of GSH under hypothermic and hyperthermic conditions, human normal liver HL-7702 cells have stronger abilities to fight against temperature stress than human liver carcinoma HepG2 cells. Hypothermia and hyperthermia can also improve the drug resistance of *cis*-dichlorodiamineplatinum(ii) (DDP)-resistant HepG2/DDP cells. The **CyO-Dise** probe has been employed to image GSH concentration changes in HepG2 and HepG2/DDP xenografts on nude mice. With the adjuvant therapy effects of hypothermia and hyperthermia, the chemotherapy drug DDP exhibits good ability for the treatment of HepG2 and HepG2/DDP xenografts. The above applications make our probe a potential new candidate for the accurate diagnosis of cancer and efficacy evaluation of treatment.

## Abstract 2

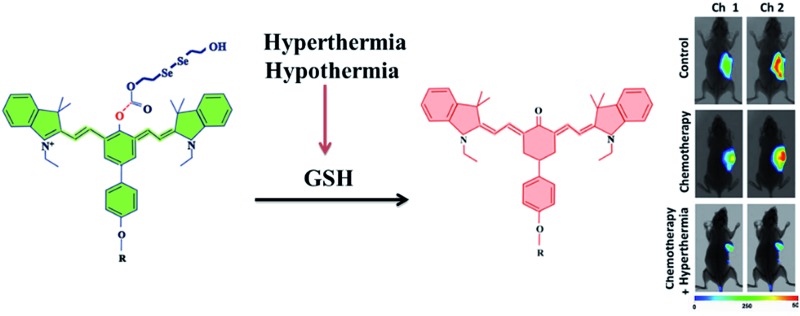
A ratiometric fluorescent probe for imaging and quantifying concentration fluctuations and anti-apoptotic effects of GSH under hypothermia and hyperthermia in HepG2 and HepG2/DDP xenografts.

## Introduction

The body temperature of mammals needs to be kept constant. Organisms often suffer from environmental temperature stress, including hyperthermia and hypothermia. Hyperthermia is a state within the temperature range of 39–50 °C,^1^ which invokes a series of cellular responses, involving metabolic pathways, inflammation reactions and apoptosis processes.^2^ Moreover, hyperthermia can induce the over-generation of intracellular reactive oxygen species (ROS), such as O_2_^–^˙ and H_2_O_2_.^3^ The overproduced ROS will lead to oxidative damage to proteins, lipids, and nucleic acids. Therefore, hyperthermia can cause oxidative stress and result in cell apoptosis.^4^ To eliminate the excessive ROS, normal cells possess strong antioxidant defense systems, such as antioxidant enzymes, which produce reductive substances and antioxidant proteins. However, cancer cells lose most of the antioxidant capacity; they are more susceptible to heat injury than normal cells.^5,6^ The hypothermia state of an organism is usually within the range from 4–32 °C.^7^ Hypothermia affects the modulation of the cell cycle, metabolism, transcription, and translation.^8^ Hypothermia has been utilized to provide protection of nerve tissue from ischemia/reperfusion injury,^9^ however, long-term hypothermia will cause some complications, such as respiratory blockades, heart failure, and infection.^10^ Similar to hyperthermia, hypothermia can also induce the over-release of ROS, which is the main factor for cell apoptosis or necrosis.^11,12^ Although both hyperthermia and hypothermia can bring oxidation stress to an organism, the key point of ROS elimination has been attributed to the anti-oxidation of biothiols.^13^

Glutathione (GSH) is the most abundant intracellular non-protein thiol for cellular antioxidant defense systems. GSH serves as the key mediator in many cellular events, including the maintenance of intracellular redox activity, xenobiotic metabolism, intracellular signal transduction, and gene regulation.^14^ The overall concentration of GSH in cytosol ranges from 0.5 to 15 mM, depending on the cell type, with an overall ratio of GSH (reduced form)/GSSG (oxidized form) ranging from 30 : 1 to 100 : 1.^15,16^ Owing to the high concentrations and the irreplaceable performance of GSH in cells, we reason that GSH must play a vital role in the control of redox homoeostasis during the hypothermia or hyperthermia process. We strive to establish the relationship between the behaviour of GSH and the cell state when organism suffers from hypothermia or hyperthermia.

The traditional methods that have been developed to determine the intracellular GSH concentration in the hypothermia or hyperthermia process are the enzymatic recycling method and electrochemical method.^13^ However, these methods are time consuming and need tedious sample pre-treatments. It is challenging to achieve *in situ* and real-time detection in living cells. Fluorescent probes are indispensable tools for bioimaging and detection, because this technology directly allows non-invasive examination of biological living samples *in situ* and offers various parameters at the biomolecular level with high spatial and temporal resolution.^17–20^ Fluorescent probes based on small molecule dyes are of particular significance due to their small size, the possibility to finely tune their properties, and the ease of chemical modification.^21–24^ Fluorescent probes for the detection of GSH have been elegantly developed.^25–27^ These presented probes are mainly designed based on the strong nucleophilic properties of GSH, such as Michael addition^28,29^ and halogen nucleophilic substitution,^30,31^ as well as the cleavage of sulfonamide,^32^ sulfonate ester,^33^ nitroazo ether,^34^ the selenium–nitrogen bond,^35,36^ and disulfide.^37^ However, no fluorescent probe has been reported to quantify the fluctuation of GSH in living cells and *in vivo* under hyperthermic or hypothermic stress. Additionally, it is also required to explore new detection mechanisms for intracellular GSH. We now attempted to develop a fluorescent probe based on a new detection mechanism for the quantification and evaluation of the anti-apoptosis effect of GSH in cells and *in vivo* under hypothermic and hyperthermic conditions.

**Scheme 1:**
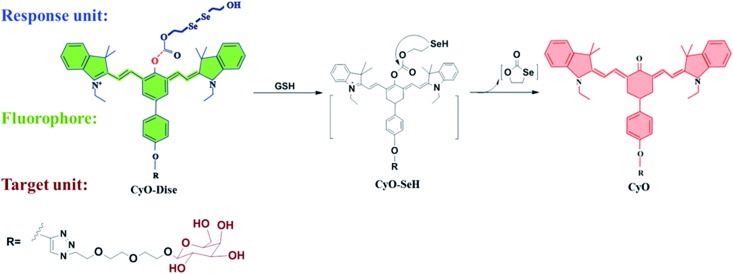
The structure of **CyO-Dise** and proposed selenium–sulfur exchange reaction through an intramolecular cyclization reaction.

Herein, we designed and synthesized a ratiometric near-infrared (NIR) fluorescent probe, **CyO-Dise**, for direct qualitative and quantitative detection of GSH fluctuations during hyperthermia and hypothermia processes in living cells and *in vivo* (Scheme 1). The detection mechanism was inspired by the selenium–sulfur exchange reaction. The probe **CyO-Dise** was composed of three moieties. The ketone cyanine fluorophore (**Keto-Cy**) was modified with the response unit bis(2-hydroxyethyl) diselenide *via* an ester linkage and targeting moiety d-galactose through a click reaction. The intracellular GSH triggered the rearrangement of the π-conjugated system in the fluorophore, which resulted in a fast ratiometric fluorescence response. As far as we know, **CyO-Dise** is the first fluorescence tool for imaging and quantifying the anti-apoptosis effects of GSH in cells and *in vivo* under hypothermic and hyperthermic conditions.

## Results and discussion

### Design and synthesis of the **CyO-Dise**

Intracellular GSH is maintained at millimolar concentrations (0.5–15 mM).^38^ The concentrations are generally higher than those of fluorescent probes (mainly ∼ μM) in living cells. Therefore, most of the reported fluorescent probes failed to qualitatively determine intracellular GSH. To accurately evaluate the fluctuations of GSH concentration during hypothermia and hyperthermia, we strived to develop a new fluorescent probe for the detection of GSH in cells. To qualitatively analyze GSH concentration at the millimolar level, the new probe should respond to changes of GSH levels with desirable reaction kinetics and thermodynamics. Taking the above factors into account, we were inspired by the selenium–sulfur exchange reaction.^39^ According to our previous work,^40^ we introduced a more reactive ester bridge instead of an amide junction in order to improve the reaction kinetics and thermodynamics of diselenide (R–Se–Se–R) towards GSH (Scheme 1). To verify this hypothesis, we computationally evaluated the detailed free energy barrier of the activation processes by GSH *via* molecular dynamics simulations and first-principles quantum mechanical/molecular mechanical free energy calculations. The calculation method is described in the ESI.† The computational results clearly revealed the corresponding free energy profile of the exchange reaction through the intramolecular cyclization reaction (Scheme S2 and Fig. S3†). From all of the molecules, the free energy barrier of the molecule containing a reactive ester bridge was calculated to be 49.6 kJ mol^–1^, while the molecule including an amide junction offered a high free energy barrier of 129.1 kJ mol^–1^. On the basis of the calculated free energy profiles, the reaction pathway of the ester bridge could be preferentially triggered by GSH. NIR absorption and emission profiles can maximize tissue penetration and minimize the absorbance of heme in hemoglobin and myoglobin, water, and lipids.^41,42^ Ratiometric probes can avoid various interference factors such as uneven loading or the inhomogeneous distribution of the fluorescent probes in cells.^43^ Integration of the response modulator into the NIR ketone cyanine scaffold could achieve a ratiometric fluorescence signal, which provides a precise and quantitative chemical tool with high sensitivity and inherent reliability.^44^ As shown in Scheme 1, the detection of GSH triggered the selenium–sulfur switch reaction, and the ester bridge accelerated the nucleophilic addition reactivation of the –SeH group, releasing the fluorophore. The rearranged polymethine π-electron system modulated a blue shift in the emission spectrum,^45^ which made our probe **CyO-Dise** a suitable chemical tool for quantifying the changes of GSH concentration in living cells. Taking advantage of the asialoglycoprotein receptor (ASGP-R), which specifically expresses on the plasma membrane of mammalian hepatocytes and selectively accepts the terminal galactose residues on desialylated glycoproteins,^46–48^ we introduced the galactose-terminated ligand here to obtain the targeting capability of **CyO-Dise** for hepatocytes *in vivo*.

The synthesis of the probe **CyO-Dise** is shown in Scheme S1.† The fluorophore **Keto-Cy** was derived from heptamethine cyanine (**Cy**) in our lab.^40^ After integrating acetyl-d-galactopyranoside into the fluorophore *via* the click chemistry reaction, the obtained compound **CyO-R′** was next treated with triphosgene. When the solvent was blow-dried in a nitrogen stream, bis(2-hydroxyethyl) diselenide was proportionally added to the reaction system. After hydrolyzing the acetyl, the probe **CyO-Dise** was finally yielded. All of the details of the synthesis are described in the ESI.†

### Spectral properties and selectivity of **CyO-Dise**

The spectroscopic properties of our probe **CyO-Dise** were investigated under simulated physiological conditions (10 mM HEPES, pH 7.4). Upon the addition of GSH, the maximum absorption wavelength shifted from 788 nm (*ε*_788 nm_ = 2.1 × 10^4^ M^–1^ cm^–1^) to 530 nm (*ε*_530 nm_ = 2.19 × 10^4^ M^–1^ cm^–1^) accompanied by a color change from green to red (Fig. S1a†). The fluorescence spectrum displayed a remarkable blue-shift from 785 nm (*Φ*_785 nm_ = 0.04) to 615 nm (*Φ*_615 nm_ = 0.13) in maximum emission wavelength (Fig. S1b and c†). The ratio of fluorescence intensity (*F*_615 nm_/*F*_785 nm_) was positively correlated with GSH concentration (Fig. S1d†) and **CyO-Dise** could selectively detect GSH without interference by other species (Fig. S1f†). As shown in Fig. S1–S5,† our probe was a good candidate for the qualitative and quantitative detection of GSH in cells and *in vivo*.

### Endogenous GSH changes under hypothermic and hyperthermic conditions

**Fig. 1:**
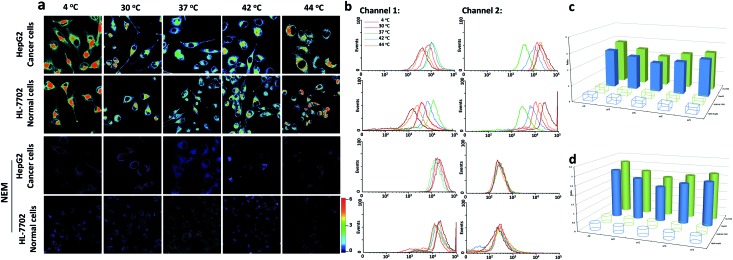
Real-time GSH quantification with **CyO-Dise** (10 μM) in HepG2 and HL-7702 cells under hypothermic (4 °C and 30 °C) and hyperthermic (42 °C and 44 °C) conditions for 30 min with or without 5 mM NEM treatment. (a) Pseudo-color ratio images. (b) Flow cytometry analysis. (c) Histograms of average ratio intensities in (a). (d) Corresponding mean ratio intensity in (b). The data are shown as mean (±s.d.) (*n* = 7).

Since our probe **CyO-Dise** had exhibited good sensitivity and selectivity towards GSH, we further investigated the potential utilization of **CyO-Dise** for the detection of GSH level changes in living cells. To assess intracellular concentrations of GSH, a human normal liver cell line (HL-7702 cells) and human hepatocellular liver carcinoma cell line (HepG2 cells) were selected as cell models. Prior to cell tests, MTT assays were performed to check the cytotoxicity of **CyO-Dise**. The high cell viability of **CyO-Dise** indicated that the probe displayed low cytotoxicity to living cells (Fig. S7†). Cell imaging experiments were performed using laser scanning confocal microscopy. All of the tested cells were incubated with 10 μM **CyO-Dise** for 5 min at 37 °C before imaging. The ratiometric fluorescence images was constructed *via* two fluorescence collection windows, channel 1 from 740 to 810 nm (*λ*_ex_ = 635 nm), and channel 2 from 580 to 630 nm (*λ*_ex_ = 515 nm). Pseudo-color ratio images were reconstructed to indicate the ratio of the emission intensity of channel 2 images and channel 1 images at the same time point. Before imaging, the cells were set at 4 °C and 30 °C for 30 min as severe and mild hypothermia states, respectively. Also, the cells were pretreated at 40 °C and 44 °C for 30 min as mild and severe hyperthermia states, respectively. As shown in Fig. 1a, either HepG2 or HL-7702 cells showed increased fluorescence intensity signal ratios after being kept in hypothermia and hyperthermia states. Under different temperature conditions, the signal intensities were obtained in the order: 4 °C > 44 °C > 30 °C > 42 °C > 37 °C. After calculations using the regression equation in Fig. S1d,† we obtained concentrations of GSH in HL-7702 and HepG2 cells (Table S1†). The results demonstrated that the cells could generate GSH in order to protect cells from temperature stress. Compared with HepG2 cells, HL-7702 cells displayed a higher ratio of fluorescence signal intensity under hypothermic or hyperthermic conditions (Fig. 1a). The results illustrated that different kinds of cells had different abilities to generate GSH. If the cells were pretreated with 5 mM *N*-ethylmaleimide (NEM) for 30 min in order to consume all of the GSH, almost no ratioed fluorescence signal was observed in HepG2 cells or HL-7702 cells (Fig. 1a). The results indicated that our probe could selectively image GSH fluctuations in hypothermia and hyperthermia states in living cells. Flow cytometry analysis, a kind of high-throughput assay technology, was performed to verify the above results. The results (Fig. 1b and Table S2†) were consistent with the pseudo-color ratio images results (Fig. 1a and Table S1†). The average fluorescence intensities of **CyO-Dise** under hypothermic and hyperthermic conditions in Fig. 1a and the corresponding mean ratio intensities in Fig. 1b were further shown in Fig. 1c and d. Additionally, the GSH concentrations were further measured using a Total Glutathione Assay Kit (Table S3†). The testing results were consistent with each other. All of the results revealed that the probe **CyO-Dise** could be applied as a facilitative tool to directly image GSH fluctuations in hypothermia and hyperthermia states in living cells.

### Evaluation the anti-apoptotic effects of GSH

**Fig. 2:**
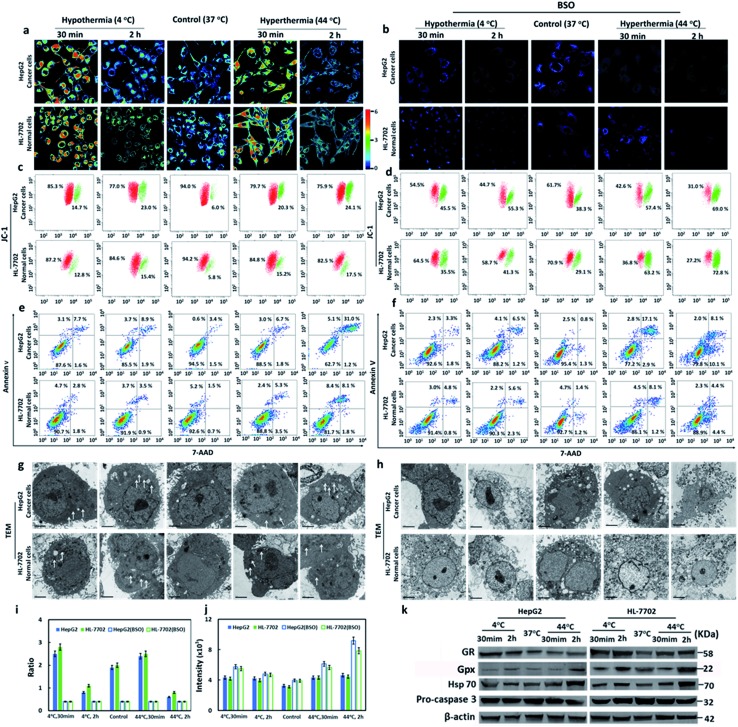
Evaluation of the anti-apoptosis effects of GSH in cells during hypothermia (4 °C) and hyperthermia (44 °C) states at time points of 30 min and 2 h. (a and b) Pseudo-color ratio images. (c and d) Mitochondrial membrane potential (Δ*Ψ*_m_) analyzed using JC-1. (e and f) Apoptosis analysis by Annexin V/7-AAD: viable cells (Annexin V–/7-AAD–), early apoptosis (Annexin V+/7-AAD–), late apoptosis (Annexin V+/7-AAD+), and necrosis (Annexin V–/7-AAD+). (g and h) TEM observation (scale bars: 2 μm). (i) Flow cytometry analysis of GSH concentrations using **CyO-Dise**. (j) Ca^2+^ concentration analysis using Fluo 4-AM. (k) Western blot analysis of GR, Gpx, Hsp70, and pro-caspase 3. Relative molecular mass is indicated on the right. β-Actin was used as a loading control. The experiments were repeated seven times (*n* = 7 per test) and the data are shown as the mean (±s.d.).

It has been reported that hypothermia and hyperthermia stimulations would induce cell apoptosis. Since our probe had been successfully applied to image the GSH concentration fluctuations during hypothermia (4 °C) and hyperthermia (44 °C), we tried to evaluate the anti-apoptotic effects of GSH in different types of cells during the hypothermia and hyperthermia processes. Cells were divided into five groups according to different treatments: 4 °C and 30 min; 4 °C and 2 h; 37 °C (as control); 44 °C and 30 min; 44 °C and 2 h. Compared with the control group, the cells in Fig. 2a at either 4 °C or 44 °C for 30 min displayed increased ratio-fluorescence signals, but those in the 2 h groups showed decreased ratio-fluorescence signals for the same kind of cell. The hyperthermic state offers higher ratio-fluorescence signals than those of hypothermia at the same time point. Upon comparison of HepG2 cells with HL-7702 cells, the ratio-fluorescence signals of HepG2 cell groups were weaker than those of HL-7702 cell whether at hypothermia or at hyperthermia (Fig. 2a). This result suggests the fact that normal cells (HL-7702 cells) could respond more to GSH changes than cancer cells (HepG2 cells) under the stresses of hypothermia and hyperthermia. The data that were obtained from laser scanning confocal microscopy were further verified using flow cytometry (Fig. 2i). We found that the concentrations of GSH first increased and then decreased during both stimulation processes. These changes of GSH concentration suggested that the intracellular GSH plays important roles in cell self-protection. Under short-term temperature stress (30 min), the mechanism of cell self-protection had a rapid response to changes in temperature stress. However, long-term stimulations (2 h) by hypothermia and hyperthermia were beyond the ability of cell self-protection. Upon incorporation into cells, cysteine is rapidly used to synthesize GSH. In order to further check the anti-apoptotic effects of GSH during hypothermia and hyperthermia, exogenous cysteine was added to increase intracellular GSH concentration. As shown in Fig. S14,† the high concentrations of GSH could evidently reduce apoptosis when the test cells suffered from temperature stress. The results showed that GSH plays anti-apoptotic roles under hypothermic and hyperthermic conditions. Glutathione reductase (GR) is a key enzyme that catalyzes the reaction of oxidized glutathione (GSSG) to the reduced form (GSH), while the elimination of GSSG by GSH mainly relies on glutathione peroxidase (Gpx).^49^ We employed Western blot analysis to examine the levels of the two enzymes in the two different types of cell (Fig. 2k and S15†). It was obvious that the HepG2 and HL-7702 cells that were treated at 4 °C or 44 °C for 30 min expressed much higher levels of GR than those cells that were treated for 2 h, while Gpx was upregulated after the 2 h treatment compared to those cells which were treated for 30 min. The enzymes within the five groups proved to have similar effects on the trends of the fluctuations of GSH concentration, which were highly consistent with the results measured with our probe.

We next confirmed the cell apoptosis that was caused by temperature stress *via* several apoptotic markers. Calcium ions (Ca^2+^) are one of the most important secondary messengers, and are involved in regulating many physiological processes in cells and tissues.^50^ In the apoptotic processes, the Ca^2+^ concentration of cytoplasm increased. We tried to assess the Ca^2+^ concentration with Fluo 4-AM *via* flow cytometry. As shown in Fig. 2j and S12,† Ca^2+^ concentrations sharply increased after hypothermia and hyperthermia as compared with the control sample. Hyperthermia led to more release of Ca^2+^ than hypothermia at the same treatment time point. The elevation degrees were positively correlated with the stimulation time, and the increased concentration in the HepG2 cells was higher than that of the HL-7702 cells under the same conditions.

The results illustrated that the decrease of GSH concentration would induce the release of Ca^2+^ into the cytoplasm, which implies mitochondrial apoptosis. Mitochondrial membrane potential (Δ*Ψ*_m_) is another biomarker in the early apoptotic stage of cells. The efflux of mitochondrial Ca^2+^ can result in a collapse of Δ*Ψ*_m_, which directly reveals cell apoptosis. Δ*Ψ*_m_ can be measured using the J-aggregate-forming lipophilic cation 5,5′,6,6′-tetrachloro-1,1′,3,3′-tetraethylbenzimidazolylcarbo-cyanine iodide (JC-1) through flow cytometry.^51^ The changes of Δ*Ψ*_m_ were detected *via* the red/green fluorescence ratio. As shown in Fig. 2c, the decreased red/green fluorescence intensity ratios were detected in all hypothermia and hyperthermia stimulation groups. As displayed in Fig. 2a and i, hyperthermia led to a greater decrease in fluorescence intensity ratios than hypothermia at the same treatment time point. The degrees of decrease in the ratios were proportional to the stimulation time. It was clear that HepG2 cells suffered a more severe decrease in fluorescence ratios than HL-7702 cells under the same conditions. The results were consistent with those received from Ca^2+^ analysis. Mitochondrial membrane potential collapse, which was caused by the efflux of Ca^2+^, resulted in the activation of apoptosis.

We also used an Annexin V/7-AAD Apoptosis Detection Kit to analyze the percentage of early apoptotic, late apoptotic, and necrotic cells. As shown in Fig. 2e, the percentage of early apoptotic and late apoptotic cells increased under hypothermic and hyperthermic conditions. However hyperthermia led to more severe cell apoptosis than hypothermia at the same treatment time point. Although the 2 h treatment resulted in more serious apoptotic damage than the 30 min treatment, HepG2 cells displayed remarkably more apoptosis and necrosis when compared to HL-7702 cells. The subcellular structure of the cells under hypothermic and hyperthermic conditions for 30 min and 2 h were observed using transmission electron microscopy (TEM). The mild swelling on the mitochondria (Fig. 2g) demonstrated that cell apoptosis occurred under hypothermia and hyperthermia conditions. Hsp70 (Heat Shock Protein 70) can protect cells from the adverse damage caused by hypothermia and hyperthermia. We analyzed the expression levels of Hsp70 in HL-7702 and HepG2 cells using Western blotting (Fig. 2k and S15c†). After stimulating hypothermia and hyperthermia, the levels of Hsp70 in the two types of cells increased. Interestingly, hyperthermia induced greater Hsp70 expression than hypothermia under the same conditions. With the passage of time, the expression levels of Hsp70 increased. However, HepG2 cells expressed lower levels of Hsp70 than HL-7702 cells. The activation of an apoptotic executor, caspase 3, was assessed using the levels of pro-caspase 3 (Fig. 2k and S15d†). Under the conditions of hypothermia and hyperthermia, the levels of pro-caspase 3 would decrease. Hyperthermia induced a greater reduction of pro-caspase 3 than hypothermia. The longer temperature stress was maintained for, the lower the level of pro-caspase 3, and the apoptosis degrees of liver cancer cells are more severe than normal liver cells. The results demonstrated caspase 3 could be activated when stimulated by hypothermia and hyperthermia. Additionally, hyperthermia was more effective on the induction of apoptosis to hepatocellular carcinoma cell lines.

Buthionine sulphoximine (BSO) is an inhibitor for gamma-glutamylcysteine synthetase (γ-GCS).^52^ The addition of BSO will reduce the cellular GSH concentrations. The faint ratio of the fluorescence signal in Fig. 2b suggested low levels of intracellular GSH. We also checked the changes of intracellular Ca^2+^ concentration, cellular Δ*Ψ*_m_, and cell apoptosis. The treatment with BSO could cause increase of Ca^2+^ concentrations (Fig. 2j). When compared with Fig. 2c, the cells in Fig. 2d provided lower red/green fluorescence ratios. The cells in Fig. 2f exhibited higher apoptosis and necrosis than the cells in Fig. 2e. The TEM images showed serious damage on cell membrane, organelles, and nuclear membrane (Fig. 2h). These findings confirmed that GSH was of critical importance for anti-apoptosis during hypothermia and hyperthermia. After comprehensively examining the above experimental results, we had reason to suggest that normal HL-7702 cells possessed a stronger ability to resist hypothermia and hyperthermia than HepG2 cancer cells.

### Mechanism of the bio-effects of GSH in drug resistance

**Fig. 3:**
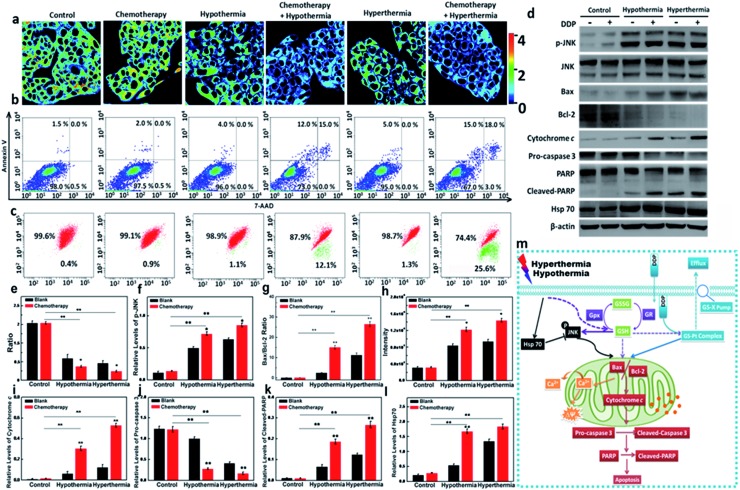
The mechanism of the anti-apoptotic effect of GSH in HepG2/DDP cells under hypothermia and hyperthermia conditions. (a) Pseudo-color ratio images. (b) Apoptosis analysis using Annexin V/7-AAD: viable cells (Annexin V–/7-AAD–), early apoptosis (Annexin V+/7-AAD–), late apoptosis (Annexin V+/7-AAD+), and necrosis (Annexin V–/7-AAD+). (c) Mitochondrial membrane potential analyzed by JC-1. (d) Western blotting analysis of p-JNK, Bax, Bcl-2, cytochrome c, pro-caspase 3, cleaved-PARP, and Hsp70. (e) Flow cytometry analysis of GSH concentrations using **CyO-Dise**. (f) A statistical analysis of p-JNK protein expression. (g) A statistical analysis of the Bax to Bcl-2 protein expression ratio. (h) Ca^2+^ concentration analysis using Fluo 4-AM. (i) A statistical analysis of cytochrome c protein expression. (j) A statistical analysis of pro-caspase 3 protein expression. (k) A statistical analysis of cleaved-PARP protein expression. (l) A statistical analysis of Hsp70 protein expression. β-Actin was used as a loading control. (m) The mechanism of bio-effects of GSH in drug-resistant cells under stimulations of hypothermia and hyperthermia. The data are shown as means (±s.d.) (*n* = 7). The differences between the data were analyzed *via* two-way ANOVA. *P < 0.05, **P < 0.01.

As one of the commonly used chemotherapeutic drugs, *cis*-dichlorodiamineplatinum(ii) (DDP) can induce DNA intrastrand crosslinks and inhibit the replication and transcription of cancer cells.^53^ Unfortunately, long-term administration can result in DDP-resistance due to the GS-X pump on the cell membrane.^54^ The efflux of DDP relies on the formation of a GSH–Pt complex. Since hypothermia and hyperthermia could induce the decrease of GSH levels, we performed additional assays to inspect whether hypothermia and hyperthermia could reduce DDP-resistance *via* the changes of GSH level. We selected the resistant HepG2/DDP cell line as test model. The DDP-resistant HepG2/DDP cells were harvested *via* a 5 month simulation by DDP.^55^ The cell model was verified *via* MTT assay, and it has a much higher IC_50_ value (27.93 μg mL^–1^) compared with 1.93 μg mL^–1^ of the HepG2 cells (Fig. S16†). We then identified the relationship between the GSH concentration and apoptosis of HepG2/DDP cells in the presence of DDP under the hypothermic and hyperthermic conditions. The apoptotic levels of HepG2/DDP cells in different therapeutic groups were measured using an Annexin V/7-AAD assay. As shown in Fig. 3b, both hypothermia and hyperthermia facilitated more apoptosis of HepG2/DDP cells than the group that was only treated with DDP. We then detected the changes of GSH in all experimental cell groups. As illustrated in Fig. 3a and e, the chemotherapeutic drug DDP hardly induced the changes of GSH concentration, while hypothermia and hyperthermia led to a significant decrease of intracellular GSH. It was noteworthy that the concentration of GSH in the cell group co-treated with DDP and hypothermia/hyperthermia presented a much lower ratio, which revealed severe apoptosis. However, exogenous cysteine treatments in Fig. S22† did not induce more apoptosis of HepG2/DDP cells than the control group, which was due to the higher GSH concentrations in the cysteine pre-treatment groups. This result indicated that the decrease of GSH resulting from hypothermia and hyperthermia could improve the DDP-resistance of HepG2/DDP cells. This mechanism of apoptosis induction was further validated in the following experiments.

As a dominant reductive species in cells, GSH protects the cells from oxidative stress in mitochondria. The changes of GSH concentration are involved in many pathological processes, such as cancer and inflammation. Therefore, hypothermia and hyperthermia have been applied to cancer adjuvant therapy. We hypothesized that the improvement of the DDP-resistance for HepG2/DDP cells benefited from the decrease of GSH concentration under the simulation of hypothermia or hyperthermia. As is known, p-JNK is a trigger of mitochondrial apoptosis, which leads to the increasing ratio of two downstream signal proteins: Bax and Bcl-2.^56^ Then Ca^2+^ stored in mitochondria effluxes into cytoplasm, which results in mitochondrial membrane potential collapse. Now the cells are becoming apoptotic. The expression of p-JNK, Bax, and Bcl-2 were examined using Western blot analysis. The changes of Ca^2+^ concentration and Δ*Ψ*_m_ were investigated *via* flow cytometry analysis. The cell model groups were set as described in Fig. 3a. Compared with the control, there was no difference in the expression levels of p-JNK (DDP-treated group, Fig. 3d and f). p-JNK could be upregulated in the cell groups under the condition of hypothermia or hyperthermia. As expected, the synergic effects between DDP and hypothermia/hyperthermia significantly promoted the upregulation of p-JNK. Similarly, the ratio of Bax and Bcl-2 were at higher expression levels with the synergic effects between DDP and hypothermia/hyperthermia (Fig. 3d and g). In terms of downstream effects, higher Ca^2+^ concentrations were detected in cytoplasm with the co-stimulation of DDP and temperature stress (Fig. 3h). The red/green fluorescence ratio of Δ*Ψ*_m_ in Fig. 3c continued to decrease with the degree of mitochondrial Ca^2+^ efflux. The results revealed that GSH concentration changes caused by temperature stress played decisive roles in improving the resistance of HepG2/DDP cells.

Moreover, cytochrome c is also a downstream effector for Bax and Bcl-2. Cytochrome c is a hydrogen carrier in the electron transport chain, which is located on the mitochondrial outer membrane.^57^ Once the mitochondrial membrane potential collapses, cytochrome c releases into cytoplasm from mitochondria, and is an apoptotic mediator to activate the apoptotic executor cleaved-caspase 3 and results in the cleavage of PARP (poly ADP ribose polymerase) in the nucleus.^58,59^ We next evaluated the expression levels of cytoplasmic cytochrome c, cleaved-caspase 3 and cleaved-PARP under the stimulating conditions. As shown in Fig. 3d and i, the level of cytochrome c in cytoplasm increased during hypothermia and hyperthermia. The addition of DDP would cause a substantial increase of cytoplasmic cytochrome c due to the synergy of hypothermia or hyperthermia. The cleaved-caspase 3 and cleaved-PARP were at higher levels in the synergy group, which is consistent with the apoptosis results tested *via* Annexin V/7-AAD assay (Fig. 3d, j and k). We also examined the Hsp70 expression in the above testing groups (Fig. 3d and l). We realized that the increase in Hsp70 levels is only dependent on the temperature stress factors, regardless of the utilization of DDP. The results might indicate that Hsp70 was activated to protect the cells from temperature stress when GSH concentrations began to decrease.

Herein, we emphasized that the stresses coming from hypothermia and hyperthermia would lead to the decrease of intracellular GSH. The mechanism of the bio-effects of GSH in drug-resistant cells during hypothermia and hyperthermia is shown in Fig. 3m. For those drug-resistant cells that are involved in the participation of GSH, such as HepG2/DDP cells, the low level of GSH would avoid the efflux of DDP. Therefore, the adjuvant therapy *via* hypothermia and hyperthermia would help to improve the cell resistance. For the mitochondrial apoptosis pathway, the low concentration of GSH triggered the increasing expression of p-JNK, which resulted in the increasing ratio of Bax/Bcl-2. Next, the efflux of Ca^2+^ in mitochondria resulted in mitochondrial membrane potential collapse. After cytochrome c was released into cytoplasm from mitochondria, the apoptotic executor cleaved-caspase 3 was activated. Finally, the cleavage of PARP occurred in the nucleus.

### Therapy efficacies of hypothermia and hyperthermia for HepG2 xenografts

**Fig. 4:**
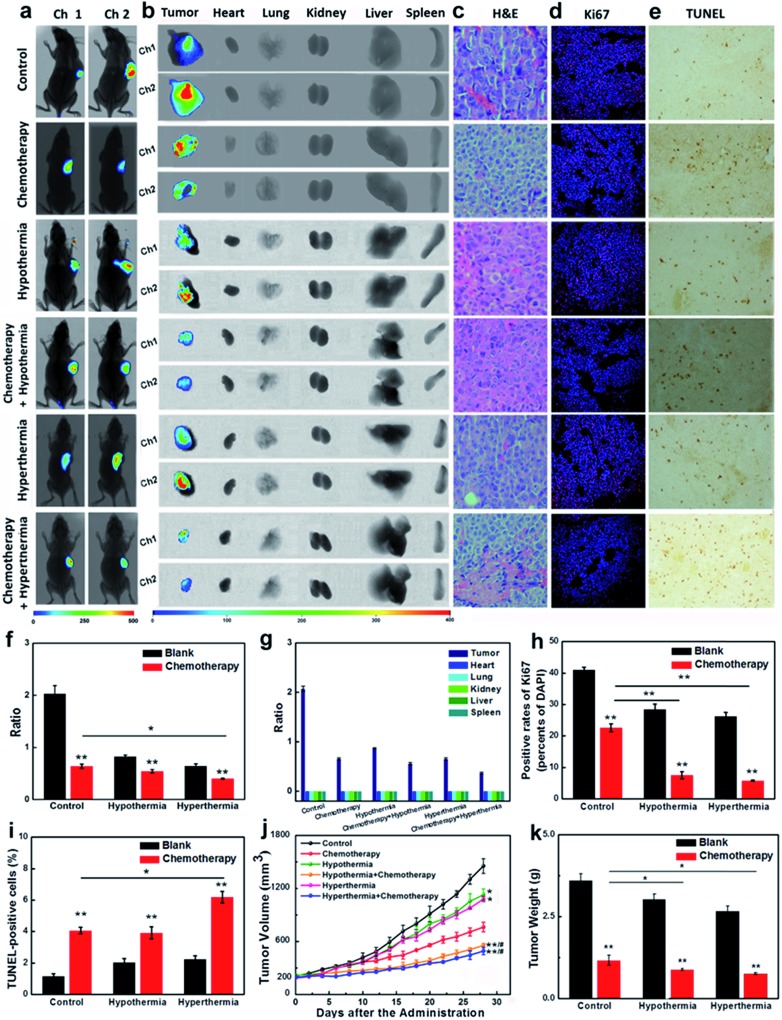
The evaluation of GSH efficacy in HepG2 subcutaneous tumor xenografts. (a) *In vivo* imaging for 30 min after tail vein injection of a single-dose 0.2 mL of **CyO-Dise** (DMSO/saline 1 : 1/v/v) (*n* = 7 per group). (b) *Ex vivo* imaging of GSH in separated organs (lung, heart, liver, kidney, spleen) and tumors sacrificed from (a). (c) Representative slides of H&E-stained tumors sacrificed from (a). Magnification: ×400. (d) Immunofluorescence staining of proliferation markers Ki67 (red channel: *λ*_ex/em_ = 635/670–770 nm) with anti-Ki67 mAb (Alexa Fluor 647 Conjugate) and nucleus (blue channel: *λ*_ex/em_ = 405/410–490 nm) with DAPI of tumor sections. Scale bar: 50 μm. (e) TUNEL staining of tumor sections. Magnification: ×100. (f) Average ratio intensity values of (a). (g) Ratio analysis of corresponding organs in (b). (h) A statistical analysis of the data derived from (d). (i) A statistical analysis of the data derived from (e). (j) Tumor sizes, where the calculation of the volume followed the formula: volume = length × width^2^ × 0.5. (k) Tumors mean weights. The error bars shown in the figures represents the mean ± s.d. The differences between the data were analyzed *via* two-way ANOVA except for tumor sizes. *P < 0.05, **P < 0.01. The differences between tumor sizes were analyzed *via* one-way ANOVA. *P < 0.05, **P < 0.01 *vs.* control group. ^#^ *P* < 0.05, ^##^ *P* < 0.01 *vs.* chemotherapy group.

Encouraged by the results that the stimulations of hypothermia and hyperthermia could induce cancer cell apoptosis *via* decreasing the concentration of GSH, we next strived to investigate the adjuvant therapy efficacies of hypothermia/hyperthermia, as well as synergistic therapy efficacies of the chemotherapy drug and temperature stress. HepG2 xenografts were established in nude mice until the tumor volumes typically reached around 200 mm^3^. Then the tumor-bearing mice were divided into six groups (Fig. 4, for details, see ESI†). The therapy groups were given 28 day chemotherapy, hypothermia/hyperthermia, and collaborative treatments, respectively. We first measured the GSH concentrations in different test groups using an *in vivo* imaging system *in vivo* and *ex vivo*. After intravenous injection for 30 min, our probe **CyO-Dise** dominantly accumulated at cancer lesions, displaying a good targeting capability of our probe for HepG2 tumors. As shown in Fig. 4a, b, f and g, hypothermia and hyperthermia led to a decrease of intracellular GSH levels. However, the synergistic therapy of the chemotherapy drug DDP and temperature stress definitely reduced the concentrations of intracellular GSH. The results indicated that hypothermia and hyperthermia could enhance the synergistic efficacies with DDP *via* lowering the concentrations of intracellular GSH.

The changes of pathological morphology were examined using H&E sections (Fig. 4c), Ki67 immunofluorescence (Fig. 4d), and TUNEL staining (Fig. 4e). H&E sections illustrated that the synergistic therapy with hyperthermia and DDP completely curbed the development of live cancer cells. Cancer cells began to differentiate, and the bile duct had already been observed. Ki67 is a cell proliferation antigen, and the positive rate of Ki67 represents the active level of cell proliferation. The inhibition of Ki67 expression in Fig. 4d and h implied that the synergistic therapy with temperature stress and DDP offered more satisfactory efficacies than other groups. TUNEL staining directly reflects the cleavage of DNA and it can be used to evaluate apoptosis. As shown in Fig. 4e and i, hypothermia and hyperthermia contribute to cell apoptosis during the chemotherapy treatment of cancer. The sizes of tumors and the body weights of mice were assessed every two days during a 28 day period (Fig. 4j and k). According to our experimental results, hypothermia and hyperthermia would lead to a decrease in intracellular GSH concentrations, and then induced cell apoptosis. Moreover, the physical therapy could effectively enhance the efficacies of chemotherapy drugs, such as DDP. However, in terms of the overall treatment efficacies and easy operation, we would give priority to hyperthermia as one of the cancer adjuvant therapies.

### Therapy efficacies of hypothermia and hyperthermia for HepG2/DDP xenografts

**Fig. 5:**
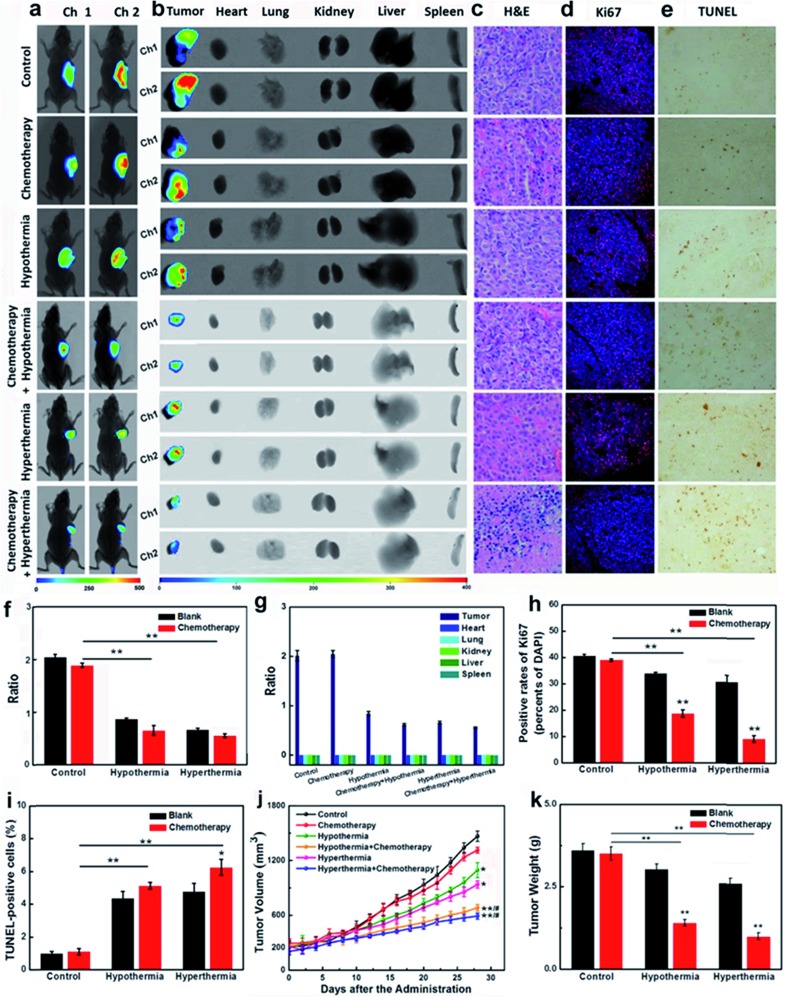
The evaluation of GSH efficacy in HepG2/DDP subcutaneous tumor xenografts. (a) *In vivo* imaging for 30 min after tail vein injection of a single-dose 0.2 mL of **CyO-Dise** (DMSO/saline 1 : 1/v/v) (*n* = 7 per group). (b) *Ex vivo* imaging of GSH in separated organs (lung, heart, liver, kidney, spleen) and tumors sacrificed from (a). (c) Representative slides of H&E-stained tumors sacrificed from (a). Magnification: ×400. (d) Immunofluorescence staining of proliferation markers Ki67 (red channel: *λ*_ex/em_ = 635/670–770 nm) with anti-Ki67 mAb (Alexa Fluor 647 Conjugate) and nucleus (blue channel: *λ*_ex/em_ = 405/410–490 nm) with DAPI of tumor sections. Scale bar: 50 μm. (e) TUNEL staining of tumor sections. Magnification: ×100. (f) Average ratio intensity values of (a). (g) Ratio analysis of corresponding organs in (b). (h) A statistical analysis of the data derived from (d). (i) A statistical analysis of the data derived from (e). (j) Tumor sizes, where the calculation of the volume followed the formula: volume = length × width^2^ × 0.5. (k) Tumor mean weights. The error bars shown in the figures represent the mean ± s.d. The differences between the data were analyzed *via* two-way ANOVA except for tumor sizes. *P < 0.05, **P < 0.01. The differences between tumor sizes were analyzed *via* one-way ANOVA. *P < 0.05, **P < 0.01 *vs.* control group. ^#^ *P* < 0.05, ^##^ *P* < 0.01 *vs.* chemotherapy group.

In the course of cancer therapy, the resistance and heterogeneity of cancer cells are challengeable problems, which need to be urgently settled. The synergistic therapy of a chemotherapy drug and temperature stress had been proven to be successful for the inhibition of the resistant HepG2/DDP cell line (Fig. 3). The nude mice model bearing HepG2/DDP xenografts were divided into six groups for our experiments (for details, see the ESI†). As shown in Fig. 5a, b, f and g, the testing groups of mice with hypothermia and hyperthermia showed a decrease in GSH concentrations. Although chemotherapy drug DDP seldom influenced the changes of the GSH levels, the co-therapy groups provided a more significant decrease of intracellular GSH, which was the pivotal point in the improvement of cancer resistance.

The H&E sections, the expressions of Ki67 and the DNA cleavage of our testing groups were investigated. Pyknosis was observed in the synergistic therapy of hyperthermia and DDP indicated good therapeutic effects on HepG2/DDP-bearing nude mice (Fig. 5c). The immunofluorescence of Ki67 showed that the adoption of temperature stress or chemotherapy alone could not desirably inhibit the proliferations of HepG2/DDP, unless using synergistic therapies, especially with hyperthermia accompanying the administration of DDP (Fig. 5d and h). The TUNEL staining illustrated DNA cleavage with the adjuvant therapies of hypothermia and hyperthermia (Fig. 5e and f). The tumor sizes, body weights and tumor weights were also recorded (Fig. 5j and k). The results displayed that the growth of cancer lesions could be efficaciously inhibited by the synergistic therapies. The triple therapeutics (GSH + chemotherapy + hypothermia/hyperthermia treatment) showed higher GSH concentrations and unsatisfactory therapeutic effects (Fig. S23†). In one word, when the cell suffered from hypothermia or hyperthermia, the decreased GSH concentration in HepG2/DDP cells would be beneficial to improving the drug resistance.

## Conclusions

We have developed a NIR radiometric fluorescent probe **CyO-Dise** for the evaluation of the anti-apoptotic effects of GSH in living cells and *in vivo*. The probe is composed of fluorophore cyanine, response unit bis(2-hydroxyethyl) diselenide, and targeting moiety d-galactose. Based on the selenium–sulfur exchange reaction, **CyO-Dise** can detect GSH within 35 s. With the help of **CyO-Dise**, we find that intracellular GSH plays important roles in anti-apoptosis when HepG2 and HL-7702 cells are faced with hypothermia and hyperthermia. The short-term temperature stress can cause increase in cellular GSH concentrations, while the long-term temperature stress can result in an abnormal decrease in the levels of cellular GSH. HepG2 cells (human hepatocellular liver carcinoma cell line) have a lower ability to resist risks of temperature stress than that of HL-7702 cells (human normal liver cell line). Moreover, hypothermia and hyperthermia can be used to improve drug resistance of the HepG2/DDP cells *via* reducing drug efflux and activating the mitochondrial apoptosis pathway. The probe has been successfully used to image the GSH levels of HepG2 and HepG2/DDP xenografts *in vivo*. The adjuvant therapy effects of hypothermia and hyperthermia have been applied to the therapies of HepG2 and HepG2/DDP xenografts. The synergistic therapy of the chemotherapy drug and temperature stress had been proven to be efficacious for the inhibition of cancer growth. We believe that our proposed strategy can be of benefit for the development of new chemical tools in the accurate diagnosis of cancer and evaluation of the efficacy of treatment.

## Conflicts of interest

There are no conflicts to declare.

## Animal Testing Statement

All experimental procedures were conducted in conformity with institutional guidelines for the care and use of laboratory animals, and protocols were approved by the Institutional Animal Care and Use Committee in Binzhou Medical University, Yantai, China. Approval Number: No. BZ2014-102R.
