# Supplementary material for: A ratiometric fluorescent probe for imaging and quantifying anti-apoptotic effects of GSH under temperature stress
Source: Chem Sci. 2017 Aug 11;8(10):6991–7002. doi: 10.1039/c7sc02888a (PMC5642144; doi:10.1039/c7sc02888a)
Supplement: Supplementary file 1 [file SC-008-C7SC02888A-s001.pdf]

Electronic Supplementary Material (ESI) for Chemical Science.
This journal is © The Royal Society of Chemistry 2017

A ratiometric fluorescent probe for imaging and quantifying anti-apoptotic effects of GSH under temperature stress

Xiaoyue Han,[a,b] Xinyu Song,[a] Fabiao Yu,[a]* and Lingxin Chen[a]*

[a] Key Laboratory of Coastal Environmental Processes and Ecological Remediation; Research Centre for Coastal Environmental Engineering and Technology,Yantai Institute of Coastal Zone Research, Chinese Academy of Sciences, Yantai 264003, China.

[b] University of Chinese Academy of Sciences, Beijing 100049, China.

E-mail: fbyu@yic.ac.cn; lxchen@yic.ac.cn

Contents:

1. General Methods
2. Synthesis and Characterization of Compounds
3. Spectral Properties and Selectivity of CyO-Dise
4. Evaluation the Detailed Free Energy Barrier of the Activation Processes
5. Effect of pH Values and Temperature Fluctuations to CyO-Dise and CyO
6. Reaction Kinetics of CyO-Dise towards GSH
7. Reaction Mechanism Discussion between Probe CyO-Dise and GSH
8. MTT Assay for CyO-Dise
9. Bright-field Images of Fig. 1
10. GSH Concentrations in Fig.1 Measured by CyO-Dise through Cell Imaging Experiments
11. GSH Concentrations in Fig.1 Measured by CyO-Dise through Flow Cytometry Analysis
12. GSH concentrations in Fig.1 Measured by Total Glutathione Assay Kit
13. Bright-field Images of Fig.2
14. Plots of Average Ratio Intensities CyO-Dise in Fig.2a
15. Flow Cytometry Analysis of GSH Concentrations by CyO-Dise for Fig.2
16. Flow Cytometry Analysis of Ca^{2+} Concentrations by Fluo 4-AM for Fig.2
17. Transmission Electron Microscopy Observation of Cells in Fig.2
18. Evaluation of the Anti-Apoptosis Effect of GSH upon Exogenous Cysteine Under Hypothermia and Hyperthermia
19. Histograms of Protein Expression in Fig.2k
20. Determination the IC_{50} of HepG2/DDP to DDP with MTT Assay
21. Bright-field Images of Fig.3a
22. Flow Cytometry Analysis of GSH Concentrations by CyO-Dise for Fig.3
23. Flow Cytometry Analysis of Ca^{2+} Concentration by Fluo 4-AM for Fig.3
24. A Statistical Analysis Protein Expression in Fig.3
25. A Statistical Analysis of GSH Concentrations by CyO-Dise in Fig.3a
26. Evaluation of the Anti-Apoptosis Effect Of GSH in Drug-resistant Cells upon Exogenous Cysteine under Hypothermia and Hyperthermia
27. Evaluation of GSH Effiency in HepG2/DDP Subcutaneous Tumor Xenografts upon Triple Therapeutics
28. Bright-field Images of Fig.4d，Fig.5d, and Fig.S23d

29. Body weight of Tumor-bearing Nude Mice in Fig.4d，Fig.5d, and Fig. S23d
30. Fluorescent Recovery Assay
31. HPLC Data for Quantification GSH Levels
32. References

1. General Methods

Materials: The solution of the probe **CyO-Dise** (1 mM) could be dissolved in dimethyl sulfoxide (DMSO) and maintained in refrigerator at 4 °C. The purity of probe was tested on a Shimadzu LC-20AT HPLC system equipped with fluorescence and UV-vis absorption detectors. When it was used for imaging, the purity of our probe was greater than 99.89%. Stock solutions of cysteine (Cys), homocysteine (HCy), glutathione (GSH), N-acetyl-L-cysteine (NAC), selenocysteine, ascorbic acid, TrxR, Ala, Arg, Asn, Asp, Cly, Gp, His, Pro, Phe, Ser, Val, N-Ethylmaleimide (NEM), Buthionine sulphoximine (BSO), cis-Dichlorodiamineplatinum (II) (DPP) were diluted to desired concentrations when needed. All the reagents were obtained from Aladdin (USA). All other chemicals were from commercial sources and of analytical reagent grade, unless indicated otherwise. 3-(4,5-Dimethylthiazol-2-yl)-2,5-diphenyltetrazolium bromide (MTT) was purchased from Sigma-Aldrich. The following rabbit antibodies were purchased from Cell Signaling Technology (β-actin: #4970, 1:1000; caspase 3: #9662, 1:1000; PARP: #9532, 1:1000; cytochrome *c*: #1940, 1:1000; Bax: #5023, 1:1000; Gpx: #3286, 1:1000; Bcl-2: #4223, 1:1000; SAPK/JNK: #9252, 1:1000; Phospho-SAPK/JNK: #4668, 1:1000; Ki67: #12075) and the following rabbit antibodies were purchased from Abcam (Hsp70: #ab90554, 1:1000; Glutathione Reductase: #ab16801, 1:1000). The following cell dyes were purchased from Thermo Fisher Scientific (JC-1: #T3168; Fluo 4-AM: #F14201). Annexin V/7-AAD Apoptosis Detection Kit was purchased from BD Biosciences (#559763). Cell Mitochondria Isolation Kit was purchased from Beyotime to obtain cytoplasm protein. Ultrapure water was used throughout.

Instruments: Absorption spectra were obtained on Lambda 35 UV-visible spectrophotometer (PerkinElmer). Fluorescence spectra were measured by FluoroMax-4 Spectrofluorometer with a Xenon lamp and 1.0-cm quartz cells. ^{1}H and ^{13}C NMR spectra were taken on a Bruker spectrometer. High-resolution mass spectra were carried on LCQ Fleet LC-MS System (Thermo Fisher Scientific). The fluorescence images of cells were acquired using a LTE confocal laser scanning microscope (Olympus FV1000 confocal laser-scanning microscope) with an objective lens (× 40). Flow cytometry data were collected by BD Biosciences FACSAria. Ultrathin sections were cut using Leica EM UC7. Transmission electron microscope images were acquired with JEOL JEM-1400 transmission electron microscope. BALB/c mice and nude mice fluorescence and x-ray images were collected by Bruker In-vivo Imaging System. Tumor pathological sections were imaged by Nikon Model Eclipse Ci-L Microscope.

Absorption and Fluorescence Analysis: Absorption spectra were obtained with 1.0-cm glass cells. Fluorescence emission spectra were obtained with a Xenon lamp and 1.0-cm quartz cells. The fluorescence intensity were measured at $\lambda_{ex/em}$ = 710/750-830 nm and at $\lambda_{ex/em}$ = 535/570-680 nm, respectively. **CyO-Dise** (0.10 mL, 1.0 mM) was added to a 10.0-mL color comparison tube. After dilution to 10 μM with 10 mM HEPES buffers, analytes were added. The mixtures were equilibrated 2 min before measurement.

Cell Culture: HepG2 cells and HL-7702 cells were purchased from the Committee on Type Culture Collection of Chinese Academy of Sciences (Shanghai, China). HepG2 cells were incubated in DMEM (Dulbecco's Modified Eagle Medium) supplemented with 10% fetal bovine serum (FBS). HL-7702 cells were incubated in RPMI (Roswell Park Memorial Institute) 1640 Medium supplemented with 10% FBS at 37 °C under a humidified atmosphere containing 5% CO_2.

The Establishment of HepG2/DDP Cell Line: For the establishment of HepG2/DPP cell line, HepG2 cells were co-cultured with DMEM containing 25 μg /mL DDP for 24 h and then washed with PBS for three times. After seven rounds of selection, the inhibitory concentration (IC_{50}) was measured by MTT assay. Until the IC_{50} reached 27.93 μg /mL, the cells clone named HepG2/DDP cells were obtained. And the HepG2/DDP cells were maintained

in a DMEM culture medium containing 0.01 μg/ml of DDP. The culture medium was replaced with DDP-free DMEM culture medium 2 weeks before the experiment.[1]

HepG2 and HL-7702 Cell Treatments: HepG2 and HL-7702 cells were maintained at 4 °C, 30 °C, 37 °C 42 °C and 44 °C for 30 min or 2 h before experiments.

HepG2/DDP Cell Treatments:
1) Control: HepG2/DDP cells were maintained at 37 °C for 2 h.
1) Chemotherapy Treatment: HepG2/DDP cells were exposed to1 μg/mL DDP for 1 h.
2) Hypothermia Treatment: HepG2/DDP cells were maintained at 4 °C for 2 h.
3) Chemotherapy + Hypothermia Treatment: HepG2/DDP cells were exposed to1 μg/mL DDP for 1 h and then maintained at 4 °C for 2 h.
4) Hyperthermia Treatment: HepG2/DDP cells were maintained at 44 °C for 2 h.
5) Chemotherapy + Hyperthermia Treatment: HepG2/DDP cells were exposed to1 μg/mL DDP for 1 h and then maintained at 44 °C for 2 h.
6) Triple Therapeutics
ⅰ) **Cysteine + Chemotherapy + Hypothermia Treatment:** HepG2/DDP cells were treated with 100 μM cysteine for 30 min before the **Chemotherapy + Hypothermia Treatment**
ⅱ) **Cysteine + Chemotherapy + Hyperthermia Treatment:** HepG2/DDP cells were treated with 100 μM cysteine for 30 min before the **Chemotherapy + Hyperthermia Treatment**

Cell Imaging: Fluorescent images were acquired on Olympus FV1000 confocal laser-scanning microscope with an objective lens (× 40). The excitation wavelength was chosen as described in paper. Cells were plated on Petri-dishes (Φ = 20 mm) and allowed to adhere for 24 h before imaging. The probe was added to the culture plates which were filled with 1 mL fresh complete medium.

Flow Cytometry Analysis: The cells were cultured at 2.0×10^5 cells/well in 6-well plates, and then treated with probes as described in the paper. After harvest, cells were washed and suspended in fresh complete medium and analyzed by flow cytometry. For channel 1: the excitation wavelength was selected 633 nm and the collected wavelengths were selected 750 - 810 nm. For channel 2: the excitation wavelength was selected 488 nm and the collected wavelengths were selected 600 - 620 nm.

Western blot analysis: All the cell samples were treated by different compounds and washed for three times before lysis of cells. Protein extracts were prepared by suspending the cells in 200 μL RIPA lysis buffer containing 1%PMSF (Solarbio, China) and 20% PhosSTOP (Roche, Germany). Then the extracts were quantified with BCA protein assay kit (Biogot, China). After denatured, the equal amounts of protein were electrophoresed on 6 – 12% SDS-polyacrylamide gels (Bio-Rad, USA) and transferred to PVDF membranes. The membrane was incubated with 5% BSA (Sigma-Aldrich, USA) and incubated with primary antibodies overnight at 4 ^{o}C with gentle shake. A horseradish peroxidase (HRP)-conjugated secondary antibody (Cell Signaling Technology, USA) was used to mirror the quantity of proteins and signals were detected with an enhanced chemiluminescence (ECL) detection system. The results were analyzed by ImageJ to acquire the grey value of every bond.

Transmission electron microscope imaging: After treated with PA, the cell samples were fixed in the fresh 2.5% glutaraldehyde for 5 h at 4° C and post-fixed in 1% osmium tetroxide for 1.5 h. Then the samples were dehydrated

in a gradient ethanol series and infiltrated with Epon812. The samples were embedded and cured at 37° C for 12 h, 45° C for 12 h and 60° C for 24 h. Ultrathin sections were cut using Leica EM UC7 and uranyl acetate and lead citrate were used to stain the section before the observation.

Histological Experiments: Tumors from nude mice were excised. The tissues were fixed in 10% formaldehyde and embedded in paraffin. Then the paraffin masses were cut and dewaxed. The sections were dehydrated using graded ethanol series and washed by distilled water. The tumor sections were stained with hematoxylin and eosion to observe the tumor tissue structure. Immunofluorescence staining followed the dehydration. After being treated with Trition X-100 and serum, the sections were stained by Ki-67 (D3B5) Rabbit mAb (Alexa Fluor® 647 Conjugate) (Cell Signaling Technology, USA) overnight. The sections were stained by DAPI to display the nucleus and enveloped by glycerol before observation. The TUNEL experiments were carried out according DeadEnd™ Colorimetric Apoptosis Detection System (Promega, USA).

Establishment of the HepG2 and HepG2/DDP Transplanted Tumor Nude Mice: 5-week-old specific pathogen free nude mice were provided by Changzhou Cavens Lab Animal Co. Ltd. The animals were housed in individual ventilated cages and fed a SPF laboratory diet and water *ad libitum*. 2×10^6 cells were suspended in media and implanted subcutaneously into nude mice. HepG2 and HepG2/DDP xenografts were established in nude mice until the tumor volumes typically reached to about 200 mm^3. All experiments were performed in accordance with the guidelines established by the Committee of Animal Research Policy of Binzhou Medical University.

HepG2 and HepG2/DDP Xenografts Treatments:

1) Chemotherapy Treatment: When the tumor size reached 200 mm^3, DDP (0.5 mg/mL × 0.5 mL) was given by intraperitoneal injection (i.p.) every other day.

2) Hypothermia Treatment: Anesthesia with isoflurane inhalation was firstly administrated. Then the tumor temperature was decreased to 32 – 34 ^oC by ice pack onto the tumors of nude mice for 1 h every other day. Infrared thermometer was used to monitor the tumor temperature.

3) Chemotherapy + Hypothermia Treatment: When the tumor size reached 200 mm^3, DDP (0.5 mg/mL × 0.5 mL, i.p.) was given every other day. Anesthesia with isoflurane inhalation was administrated to immobilize the mouse at 1 h after the injection. The tumor temperature was decreased to 32 – 34^oC by ice pack onto the tumors of nude mice for 1 h. Infrared thermometer was used to monitor the tumor temperature.

4) Hyperthermia Treatment: Anesthesia with isoflurane inhalation was firstly administrated. A microwave device was employed to process the hyperthermia therapy above the tumor 3 cm every other day. The therapy needs a frequency of 2450 MHz microwave and a therapeutic diameter of 8 cm. The skin of tumor should be kept at 42 ^oC for 1 h. Infrared thermometer was used to monitor the tumor temperature.

5) Chemotherapy + Hyperthermia Treatment: When the tumor size reached 200 mm^3, DDP (0.5 mg/mL × 0.5 mL, i.p.) was given every other day. Anesthesia with isoflurane inhalation was administrated to immobilize the mouse at 1 h after the injection. A microwave device was employed to process the hyperthermia therapy above the tumor 3 cm every other day. The therapy needs a frequency of 2450 MHz microwave and a therapeutic diameter of 8 cm. The skin of tumor should be kept at 42^oC for 1 h. Infrared thermometer was used to monitor the tumor temperature.

6) Triple Therapeutics

ⅰ) **GSH + Chemotherapy + Hypothermia Treatment:** The nude mice were given GSH (0.13 mg/mL × 0.1 mL) by tail vein injection before the **Chemotherapy + Hypothermia Treatment**

ⅱ) **GSH + Chemotherapy + Hyperthermia Treatment:** The nude mice were given GSH (0.13 mg/mL × 0.1 mL) by tail vein injection before the **Chemotherapy + Hyperthermia Treatment**

Imaging Mice *in vivo*: A Bruker *In-vivo* Imaging System was employed to image tumor bearing nude mice. The excitation and emission wavelengths were chosen as described in paper. The mice were anesthetized prior to injection and during imaging. After *in vivo* imaging, the organs (lung, heart, liver, kidney and spleen) and tumors were excised to perform *ex vivo* imaging.

Statistical Analysis: Statistical Product and Service Solutions (SPSS) software 19.0 was used for the statistical analysis. The error bars shown in the figures represented the mean ± s.d. Differences were determined with a one-way, two-way analysis of variance (ANOVA) followed by LSD test. Statistical significance was assigned at ➢$P <$ 0.05, ➢➢$P <$ 0.01. Sample size was chosen empirically based on our previous experiences and pre-test results. No statistical method was used to predetermine sample size and no data were excluded. The numbers of animals or samples in every group were described in the corresponding figure legends. The distributions of the data were normal. All experiments were done with at least three biological replicates. Experimental groups were balanced in terms of animal age, sex and weight. Animals were all caged together and treated in the same way. Appropriate tests were chosen according to the data distribution. Variance was comparable between groups in experiments described throughout the manuscript.

2. Synthesis and Characterization of Compounds

Scheme S1. Synthetic routes for probe **CyO-Dise**.

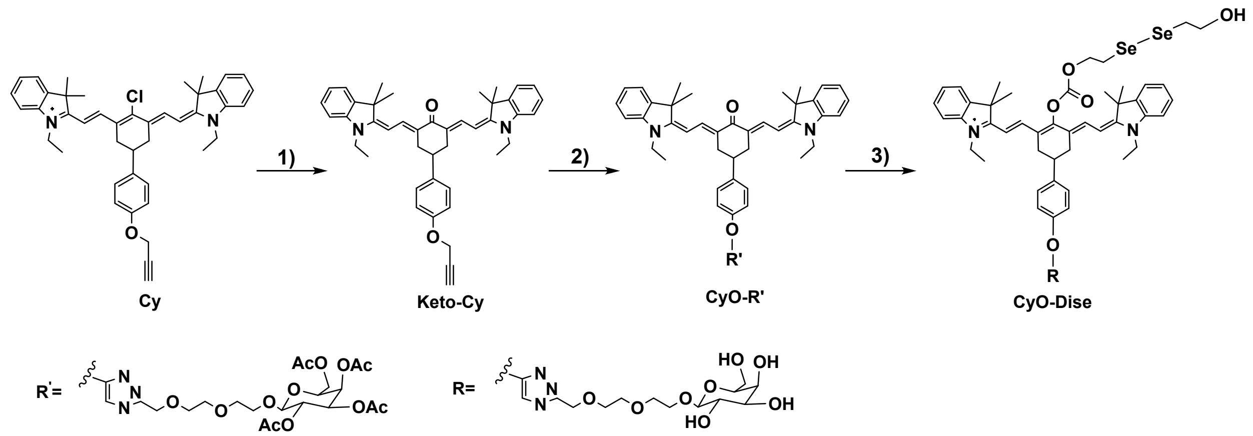

Synthesis of Keto-Cy. Cy (21.1 mg, 0.33 mmol) and AcONa (54 mg, 0.66 mmol) were dissolved in 10 mL anhydrous DMF under Ar for 5 h at 90 ^{o}C.[2] Then the mixture washed three times with saturated KI solution, and extracted by CH_2Cl_2 (50 mL × 3). All mixtures were concentrated on a rotary evaporator, and the residues were purified by silica chromatography (200 - 300 mush) eluted with dichloromethane: methanol = 15:1 (v/v) to give a dark red solid 17.4 mg. Yield: 85%. ^{1}H NMR (500 MHz, $CDCl_3$-D_1) δ (ppm): 8.27-8.24 (d, 2H), 7.37-7.36 (d, 2H),

7.19-7.16 (m, 4H), 7.04-7.03 (m, 2H),6.92-6.90 (m, 2H), 6.67-6.66 (m, 2H), 5.44-5.41 (d, 2H), 4.72-4.71 (d, 2H), 4.13-4.09 (q, 1H), 3.70-3.66 (m, 4H), 3.05-3.00 (m, 2H), 2.65-2.62 (m, 2H), 2.60 (s, 1H), 1.73 (s, 12H), 1.30-1.20 (m, 6H). ^{13}C NMR (125 MHz, $CDCl_3$-D_1) δ (ppm): 185.51, 171.12, 162.42, 156.26, 143.65, 140.00, 139.86, 136.13, 133.60, 128.15, 128.02, 127.90, 127.72, 127.62, 125.41, 122.46, 122.25, 121.83, 120.64, 115.07, 114.92, 108.20, 106.58, 91.99, 78.82, 75.58, 60.40, 55.88, 46.71, 44.09, 39.93, 37.00, 34.55, 34.03, 28.80, 28.74, 24.40, 21.07, 14.24, 12.75, 11.25. LC-MS (ESI^+): m/z $C_{43}H_{46}N_2O_2$ calcd.622.36, found [M^+] 623.42.

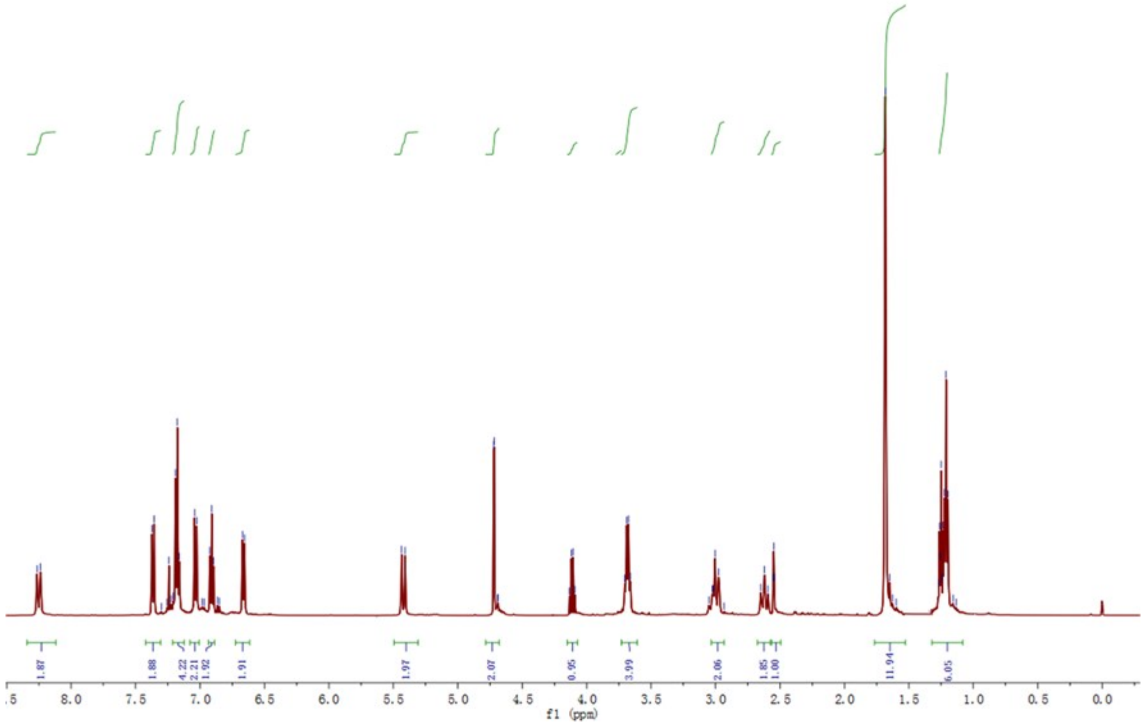

Synthesis of CyO-R′. Aqueous solutions of sodium ascorbate (0.01 M, 1 mL) and $CuSO_4 \cdot 5H_2O$ (0.01 M, 1 mL) were mixed to produce an orange suspension containing the copper (I) catalytic species under Ar atmosphere.[3] Compound **Keto-Cy** (62.2 mg, 0.10 mmol), acetyl-*D*-galactopyranoside (55.5 mg, 0.11 mmol) and DIPEA (1.5 g, 0.01 mmol) were subsequently added into 6.0 mL methanol. The mixture was further stirred for 24 h at 25 °C under Ar atmosphere. The solvent was removed in vacuum and the obtained solid residue was purified through a silica gel chromatography (200 - 300 mesh) eluted with dichloromethane: methanol= 6:1 (v/v). Compound **CyO-R′** was afforded as red solid (90 mg, yield 79.9%). ^1H NMR (500 MHz, $CDCl_3$-D_1) δ (ppm): 7.60 (s, 1H), 7.40-7.07 (m, 4H), 7.08-7.02 (m, 2H), 6.86-6.84 (m, 2H), 6.73-6.68 (m, 4H), 6.52-6.48 (m, 2H), 5.94-5.93 (m, 1H), 5.63 (s, 2H), 5.50 (d, 1H), 5.44-5.38 (m, 1H), 5.21-5.19 (m, 1H), 5.16 (s, 2H), 4.28-4.09 (m, 7H), 4.02-3.98 (m, 2H), 3.87-3.84 (t, 2H), 3.58 (t, 4H), 3.55 (d, 4H), 3.58-3.15 (m, 1H), 2.49-2.41 (q, 2H), 2.22-2.17 (q, 2H), 2.04-2.02 (m, 12H), 1.78 (s, 12H), 1.32-1.27 (t, 6H). ^{13}C NMR (125 MHz, $CDCl_3$-D_1) δ (ppm): 167.30, 150.19, 149.95, 147.74, 136.69, 136.37, 135.58, 134.34, 124.15, 123.53, 123.40, 121.03, 117.89, 117.25, 116.33, 110.53, 110.18, 105.90, 101.90, 95.21, 71.01, 70.84, 61.15, 61.00, 60.87, 60.77, 59.52, 55.48, 55.42, 51.14, 51.11, 50.22, 44.79, 42.83, 37.13, 36.80, 35.48, 32.94, 32.31, 32.25, 32.11, 32.07, 27.49, 24.90, 24.53, 23.84, 23.39, 23.37, 22.43, 15.48, 12.67, 11.20. LC-MS (ESI^+): m/z $C_{63}H_{77}N_5O_{14}$ calcd.1127.5467, found [M^+] 1128.6013.

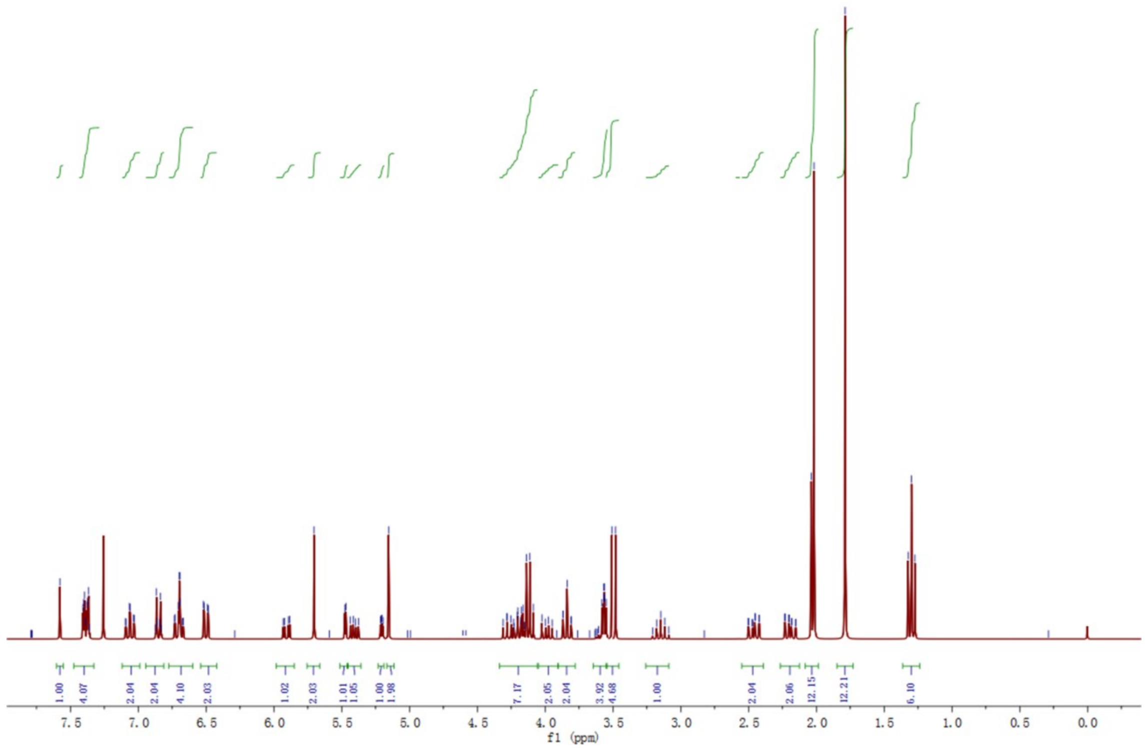

Synthesis of CyO-Dise. CyO-R′ (11.3 mg, 0.1 mmol), triphosgene (9 mg, 0.3 mmol) was dissolved in 50 mL anhydrous CH_2Cl_2 under Ar atmosphere.[4] The mixture was suspended at 0 ^oC, then 1 mL DIPEA was added. The reaction lasted for 30 min, and the color of the solution changed into green from red. After removed solvent in vacuum, the obtained residue was dissolved in 50 mL anhydrous CH_2Cl_2, and added DIPEA (1 mL) and DMAP (20 mg). bis(2-hydroxyethyl) diselenide (50.0 mg, 0.2 mmol) in CH_2Cl_2 (2 mL) was added into above mixture,[5] then the reaction mixture was stirred at 25 °C, TLC monitored the reaction until the starting material was completely consumed. The obtained solid residue was purified through a silica gel chromatography (200 - 300 mesh) with gradient eluent of CH_2Cl_2 and CH_3OH (100:0 to 85:15 v/v). The solvent was removed in vacuum, and the obtained solid was dissolved in CH_2Cl_2, filtered and evaporated to give a green solid (122 mg, yield 87%). The green solid (84.2 mg, 0.06 mmol) and potassium carbonate (1.2 mL from a 0.5 M solution in MeOH) were added in anhydrous MeOH (5 mL). The reaction mixture was stirred at 25 °C for 1 h. After neutralized with diluted hydrochloric acid (5%), the solution was extracted with CH_2Cl_2 (3 × 50 mL). The organic layer was dried with anhydrous Na_2SO_4. After concentrated under vacuum, the crude solid was purified by column chromatography (200 - 300 mesh) using CH_2Cl_2: CH_3OH (8:1 v/v) as eluent to obtain **CyO-Dise** as green solid (45 mg, 61%). ^1H NMR (500 MHz, $CDCl_3$-D_1) δ (ppm): 8.25-8.17 (d, 1H), 8.14-8.11 (m, 1H), 7.52-7.08 (m, 6H), 7.08-7.06 (m, 1H), 6.87-6.84 (m, 2H), 6.72-6.71 (m, 1H), 6.53-6.52 (m, 1H), 5.39-5.36 (q, 1H), 5.17 (d, 2H), 5.12 (t, 2H), 4.61-4.57 (m, 2H), 4.48-3.97 (m, 12H), 3.87-3.53 (m, 23H), 3.49-3.44(m, 1H), 2.55-2.52 (m, 2H), 2.28-2.23 (m, 2H), 1.86-1.79 (m, 6H), 1.41-1.21 (m, 9H), 1.18-1.07 (m, 3H). ^{13}C NMR (125 MHz, $CDCl_3$-D_1) δ (ppm): 167.30, 150.19, 149.95, 147.74, 136.69, 136.37, 135.58, 134.34, 124.15, 123.53, 123.40, 121.03, 117.89, 117.25, 116.33, 110.53, 110.18, 105.90, 101.90, 95.21, 71.01, 70.84, 61.15, 61.00, 60.87, 60.77, 59.52, 55.48, 55.42, 51.14, 51.11, 50.22, 44.79, 42.83, 37.13, 36.80, 35.48, 32.94, 32.31, 32.25, 32.11, 32.07, 27.49, 24.90, 24.53, 23.84, 23.39, 23.37, 22.43, 12.48, 11.67. LC-MS (ESI^+): m/z $C_{60}H_{78}N_5O_{13}Se_2^+$ calcd.1236.3921, found [M^+] 1236.3915.

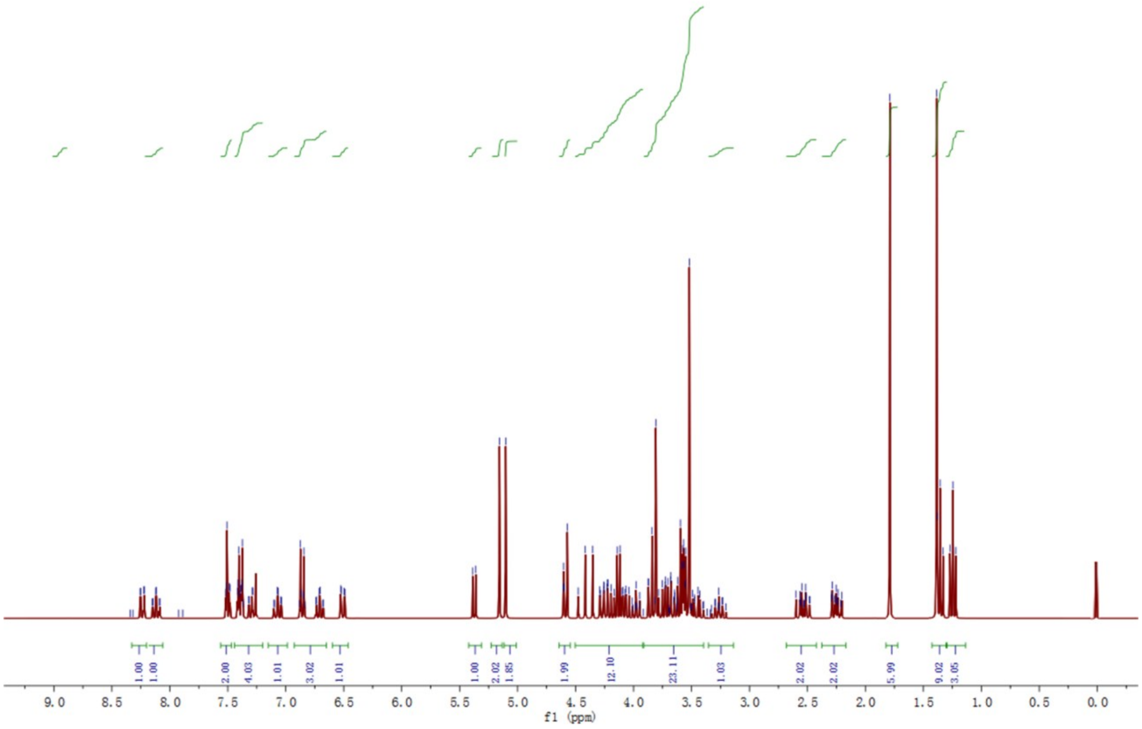

3. Spectral Properties and Selectivity of CyO-Dise

The spectroscopic properties of the probe **CyO-Dise** was investigated under simulated physiological conditions (10 mM HEPES, pH 7.4). There existed a linear relationship between logarithm of the ratio ($F_{615\ nm}/F_{785\ nm}$) and the concentrations of GSH (Fig. S1d). The regression equation was Lg($F_{615\ nm}/F_{785\ nm}$) = 0.1703 [GSH] mM - 0.6096, with r = 0.9947. The experimental detection limit was determined to be 50 μM. The theoretical detection limit was calculated to be as low as 20 μM ($3\sigma/k$), where σ was the standard deviation of blank measurement, and k was the slope of regression equation.

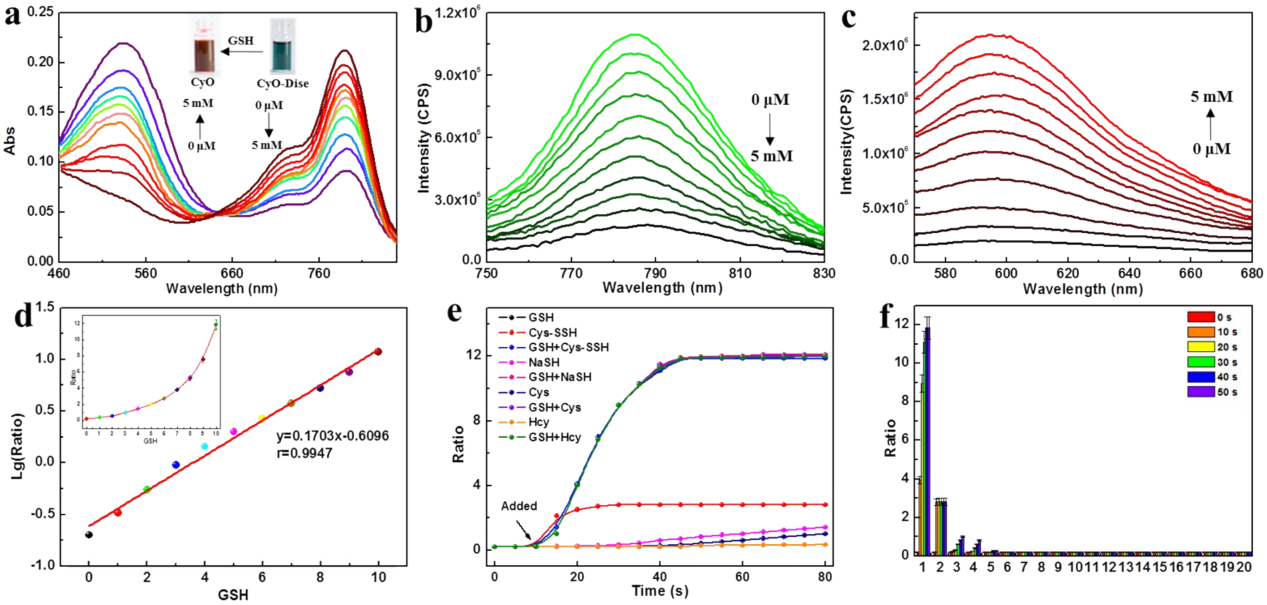

Fig. S1. Spectral properties and selectivity of **CyO-Dise**. **CyO-Dise** (10 μM) was incubated with increasing concentration of GSH (0 - 10 mM) at 37 °C in HEPES (pH 7.4, 10 mM). The spectra were recorded after 30 s. (a) Dose-dependent absorbance spectra of **CyO-Dise** towards GSH. (b) Emission spectra (λ_{ex} = 710 nm) of **CyO-Dise** towards GSH. (c) Emission spectra (λ_{ex} = 535 nm) of **CyO-Dise** towards GSH. (d) Intensity ratio ($F_{615\ nm}/F_{785\ nm}$) of **CyO-Dise** change as a function of GSH. Insert: The linear relationship between Lg

($F_{615\ nm}/F_{785\ nm}$) and GSH. (e) Time dependent fluorescent ratio $F_{615\ nm}/F_{785\ nm}$ of **CyO-Dise** towards 10 mM GSH; 5 µM Cys-SSH; 10 mM GSH +5 µM Cys-SSH; 100 µM NaHS; 10 mM GSH + 100 µM NaHS; 200 µM Cys; 10 mM GSH + 200 µM Cys; 10 µM Hcy ; 10 mM GSH + 10 µM Hcy during 0 – 80 s. (f) Time-dependent fluorescent ratio ($F_{615\ nm}/F_{785\ nm}$) response of **CyO-Dise** to various reactive species. 1, 10 mM GSH; 2, 5 µM Cys-SSH; 3, 100 µM NaHS; 4, 200 µM Cys; 5, 10 µM Hcy; 6, 10 µM selenocysteine; 7, 10 µM ascorbic acid; 8, 10 µM TrxR; 9, 10 µM N-acetyl-L-cysteine; 10, 10 µM Ala; 11, 10 µM Arg; 12, 10 µM Asn; 13, 10 µM Asp; 14, 10 µM Cly; 15, 10 µM Gp; 16, 10 µM His; 17, 10 µM Pro; 18, 10 µM Phe; 19, 10 µM Ser; 20, 10 µM Val. GSH was added at the reaction time of 10 s. The reactions were measured during 80 s. $F_{615\ nm}$: λ_{ex} = 535 nm, λ_{em} = 615 nm; $F_{785\ nm}$: λ_{ex} = 710 nm, λ_{em} = 785 nm. The experiments were repeated five times and the data were shown as mean (±s.d.).

Considering the rapid metabolism unstable nature of GSH, the reaction kinetics of **CyO-Dise** towards GSH was performed to inspect whether the probe could selectively fast respond to GSH. It should be emphasized that diselenide had been used to response cysteine persulfide (Cys-SSH). However, these two kinds of reactive sulfur species are not at an order of magnitude in living cells. The concentrations of Cys-SSH are at micromolar levels, [5] while the concentrations of GSH are at millimolar levels concentrations.[6,7] So, Cys-SSH would not lead to big interference in the GSH detection. Other biothiols including Cys-SSH, hydrogen sulfide (H_2S, provided as NaSH), cysteine (Cys), homocysteine (HCy) were also investigated their reaction kinetic with the probe. GSH and the other biothiols were respectively added at 10 s, and then the ratio-fluorescent signal response were recorded during 0 - 80 s. GSH induced ratio-fluorescent changes and reached saturation within 35 s. Cys-SSH also induce the ratio-fluorescent changes within 20 s. However, Cys-SSH could not consume all the probe. Other biothiols induced negligible ratio-fluorescent signal changes (Fig. S1e). Moreover, we investigated whether the present of biothiols could affect the detection of GSH. Exposing **CyO-Dise** to a mixture of biothiols and GSH almost yielded the same ratio-fluorescence enhancement as that only treated with GSH (Fig. S1e). The results may contribute to the higher concentration of GSH in biological organism than the other biothiols. The reaction kinetics studies indicated that our probe could be used as a real-time bioimaging tool for intracellular GSH without the interference of biothiols .

Our next efforts were made to test and verify selectivity of **CyO-Dise** towards GSH. All the tests lasted 50 s. Upon exposed to various analytes in HEPES (10 mM, pH 7.4), the probe **CyO-Dise** selectively exhibited excellent ratio fluorescence response to GSH. Other biothols (such as Cys-SSH, NaSH, Cys, Hcy), selenocysteine, ascorbic acid, thioredoxin reductase (TrxR), N-acetyl-L-cysteine and various amino acids (AAs) induced negligible interference (Fig. S1f). Reactive oxygen species, reactive nitrogen species, anions and metal ions couldn't cause any spectral changes (Fig. S2a and S2b). All the results demonstrated that **CyO-Dise** could selectively detect GSH without interference by other species.

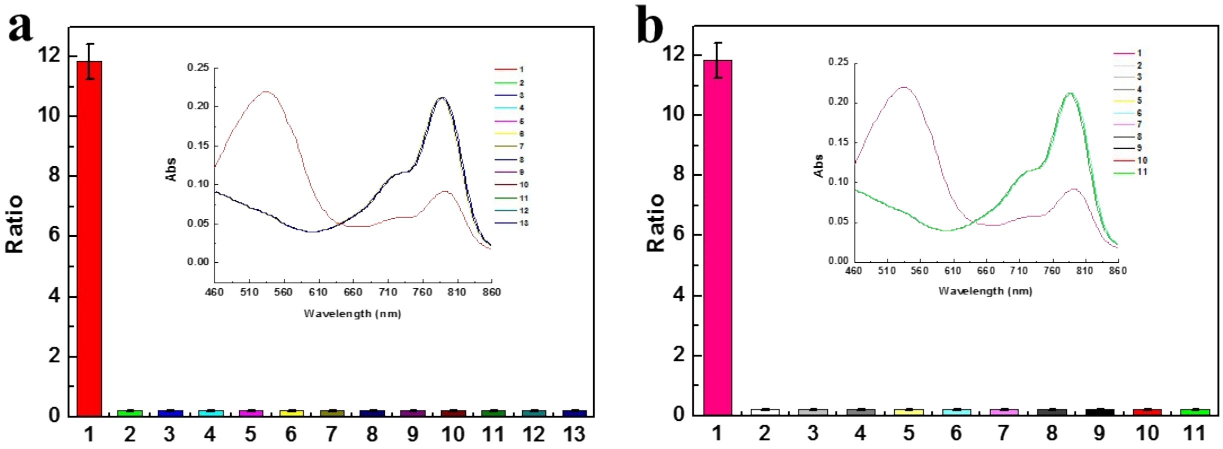

Fig. S2 Fluorescent ratio ($F_{615\ nm}/F_{785\ nm}$) response and absorbance spectra of **CyO-Dise** (10 µM) to reactive oxygen species, reactive nitrogen species, anions and metal ions in HEPES buffer (pH 7.4, 10 mM) at 37 °C. a) 1, 10 mM GSH; 2, 200 µM NO_2^-; 3, 200 µM

ONOO⁻; 4, 200 μM NO; 5, 200 μM t-BuOOH; 6, 200 μM H_2O_2; 7, 200 μM $O_2^{\cdot -}$; 8, 200 μM MeLOOH; 9, 200 μM ClO^-; 10, 200 μM CuOOH, 11, 20 μM GSNO. b) 1, 10 mM GSH; 2, 1 mM K^+; 3, 1 mM Na^+; 4, 1 mM Ca^{2+}; 5, 1 mM Mg^{2+}; 6, 1 mM Zn^{2+}; 7, 1 mM Cu^{2+}; 8, 1 mM Cl^-; 9, 1 mM Br^-; 10, 1 mM F^-; 11, 1 mM CO_3^{2-} ; 12, 1 mM $H_2PO_4^-$; 13, 1 mM Critrate. **CyO-Dise**: $F_{785\ nm}$: λ_{ex} = 710 nm, λ_{em} = 785 nm; $F_{615\ nm}$: λ_{ex} = 535 nm, λ_{em} = 615 nm. The emission and UV absorbance spectra were recorded after 60 min. The experiments were repeated five times and the data were shown as mean (± s.d.).

4. Evaluation the Detailed Free Energy Barrier of the Activation Processes

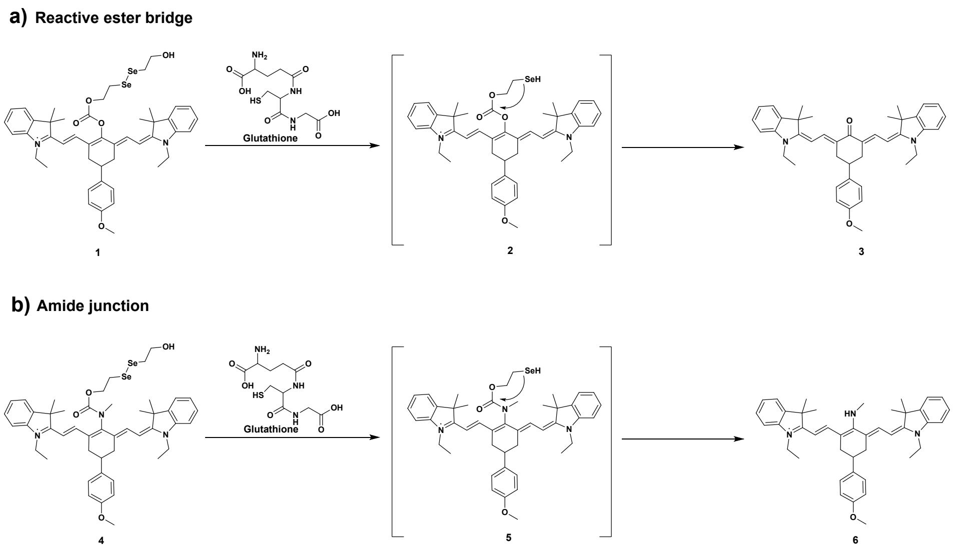

Scheme S2 Simplified structures for calculating the free energy of exchange reaction through intramolecular cyclization reaction. a) Reactive ester bridge and b) amide junction stages of the reaction pathway.

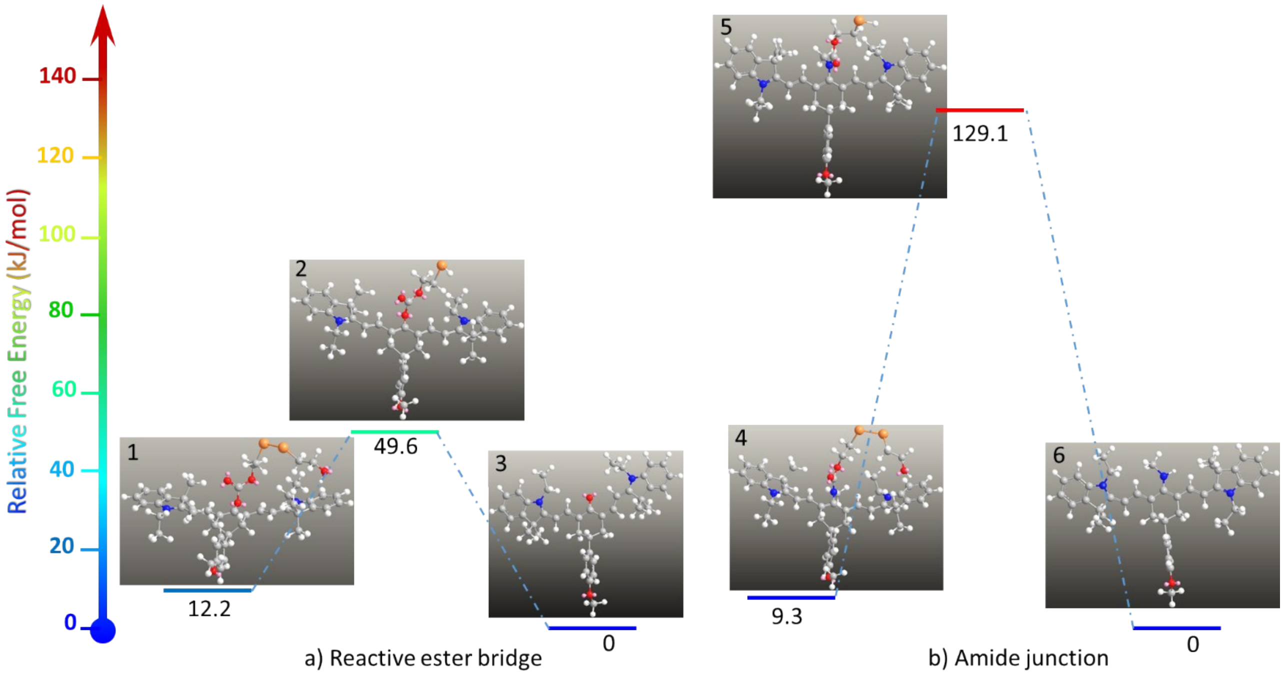

Fig. S3 Illustration of free energy profiles determined by the QM/MM-FE calculations for a) reactive ester bridge and b) amide junction stages of the reaction pathway.

The detailed reaction pathway of the exchange reaction through intramolecular cyclization reaction were explored by performing molecular dynamics (MD) simulation and first-principles quantum mechanical/molecular mechanical free energy (QM/MM-FE) calculations.[8] The geometry of all molecular structures were optimized at the HF/6-31G* level using Gaussian03.[9] The QM calculations were performed with the B3LYP functional, and the MM calculations were conducted by using a modified version of AMBER.[10] Given in parentheses are the relative free energies with the zero-point and thermal corrections for the QM subsystem. The QM/MM-FE calculations for all QM/MM-optimized geometries along the reaction path were performed at the B3LYP/6-31++G**: AMBER level. In order to simplify the molecular structure optimization and calculation processes, we replaced the galactose-terminated ligand as methoxy (Scheme S2).

5. Effect of pH Values and Temperature Fluctuations to CyO-Dise and CyO

Standard fluorescence pH titrations were performed in 10 mM HEPES solution at a concentration of 10 μM (**CyO-Dise** and **CyO**). As shown in Fig. S3a and 3b, the pH of the media hardly effected on fluorescence intensity within the range from 3.0 to 10.0. On this basis, we suggested that **CyO-Dise** would work well under physiological conditions (pH = 7.4). Standard fluorescence temperature titrations were performed in 10 mM HEPES solution at a concentration of 10 μM (**CyO-Dise** and **CyO**). As shown in Fig. S3c and 3d, the temperature of the mediums hardly effected on fluorescence intensity within the range from 4 °C to 44 °C. On this basis, we suggested that **CyO-Dise** would work well under physiological conditions (pH = 7.4).

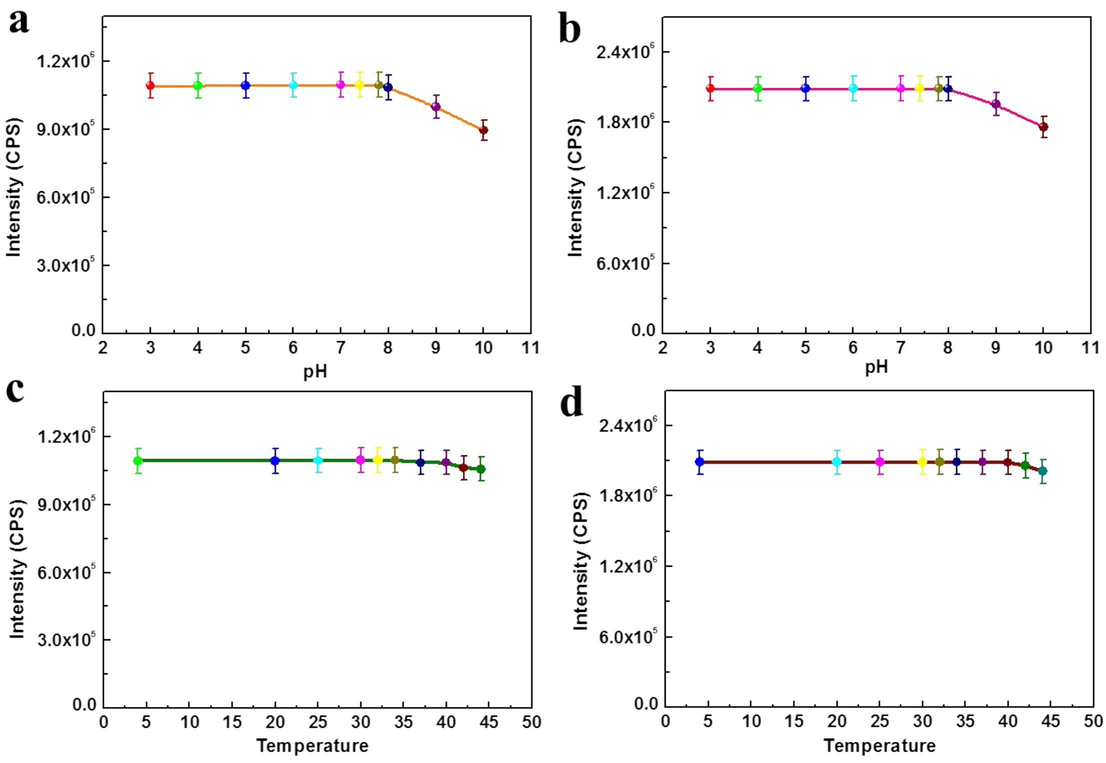

Fig. S4 Effect of pH values and temperature fluctuations. a) The fluorescence emission changes at 710 nm with the pH titration curve of **CyO-Dise** (10 μM). b) The fluorescence emission changes at 535 nm with the pH titration curve of **CyO** (10 μM). pH: 3.0, 4.0, 5.0, 6.0, 7.0, 7.4, 7.8, 8.0, 9.0, 10.0 (10 mM HEPES buffer). Effect of temperature fluctuations. c) The fluorescence emission changes at 710 nm with the temperature fluctuations curve of **CyO-Dise** (10 μM). d) The fluorescence emission changes at 535 nm with the temperature fluctuations curve of **CyO** (10 μM). Temperature: 4 °C; 20 °C; 25 °C; 30 °C; 32 °C; 34 °C; 37 °C; 40 °C; 42 °C; 44 °C (10 mM HEPES buffer). $F_{785\ nm}$: λ_{ex} = 710 nm, λ_{em} = 785 nm; $F_{615\ nm}$: λ_{ex} = 535 nm, λ_{em} = 615 nm. The experiments were repeated five times and the data were shown as mean (± s.d.).

6. Reaction Kinetics of CyO-Dise towards GSH

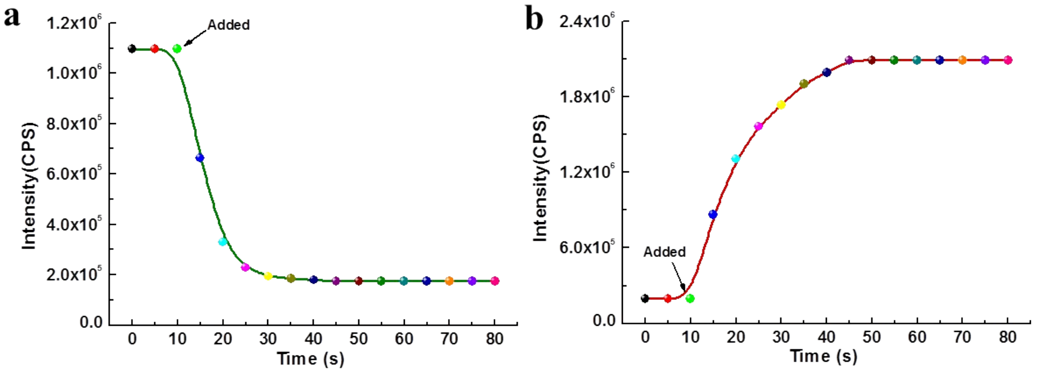

Fig. S5 Time dependent fluorescent ratio ($F_{615\ nm}/F_{785\ nm}$) of probe **CyO-Dise** (10 μM) towards GSH (10 mM) during 0 - 80 s. GSH was added at the reaction time of 10 s in 10 mM HEPES buffer (pH 7.4). The reactions were measured during 0 - 80 s at 37 ^{o}C. $F_{785\ nm}$: λ_{ex} = 710 nm, λ_{em} = 785 nm; $F_{615\ nm}$: λ_{ex} = 535 nm, λ_{em} = 615 nm. The experiments were repeated five times and the data were shown as mean (± s.d.).

7. Reaction Mechanism Discussion between Probe CyO-Dise and GSH

We had described the reaction mechanism in Scheme 1, the reduction of diselenide immediately formed an intermediate (**CyO-SeH**). Then a fast intramolecular cyclization occurred by cleavage of neighboring ester bond to release **CyO**. This proposed reaction mechanism was verified by HRMS, as shown in Fig. S6. The reaction intermediate **CyO-SeH** HRMS (ESI$^+$): m/z $C_{59}H_{77}N_5O_{12}Se^+$ calcd. 1127.4728, found [M$^+$] 1127.4756. The reaction product **CyO** HRMS (ESI$^+$): m/z $C_{55}H_{69}N_5O_{10}$ calcd. 959.5044, found [M$^+$] 959.5135.

However, there is a possibility which should be addressed, that is, the reduction of diselenide may form an intermediate **CyO-Se-SG** (Scheme S3). However, we did not detect intermediate **CyO-Se-SG** by HRMR. Maybe intermediate **CyO-Se-SG** was high reactivity or unstable. Nonetheless, the intermediate selenylsulfides of **CyO-Se-SG** could be reduced by GSH straightway to form **CyO-SeH**. Then a fast intramolecular cyclization occurs by cleavage of neighboring carbamate bond to release fluorophore. Even if our probe might follow the steps according to Scheme S3, there was also no extra consumption by the reaction.

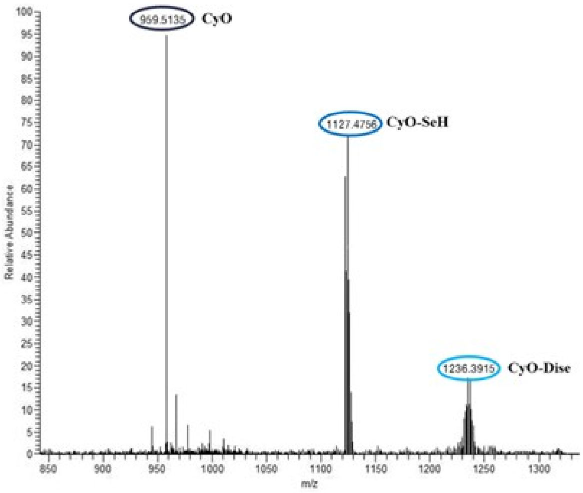

Fig. S6 HRMS of reactant **CyO-Dise**, the reaction intermediate **CyO-SeH** and the reaction product **CyO**.

Scheme S3. The possible reaction mechanism between probe **CyO-Dise** and GSH.

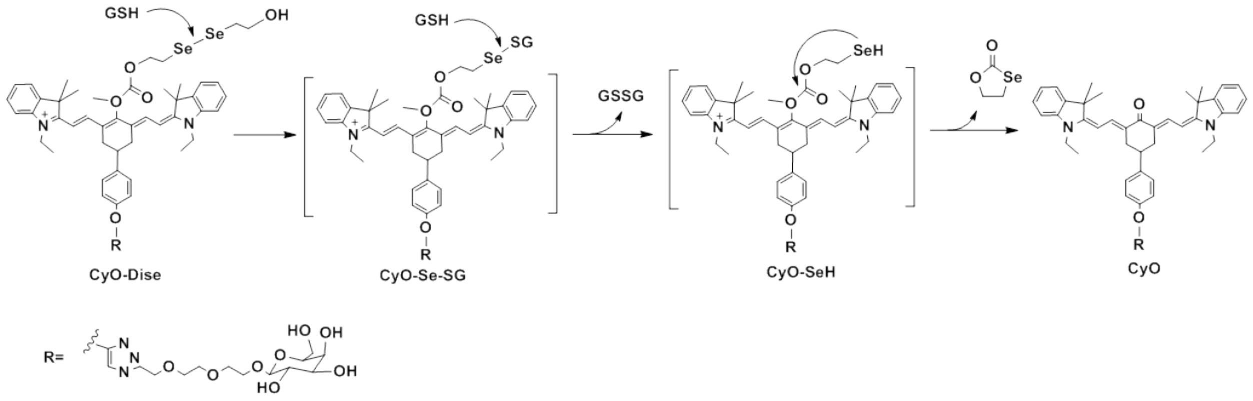

8. MTT Assay for CyO-Dise

To access the potential toxicity of **CyO-Dise**, MTT assays were carried out. HepG2 cells (10^6 cells/mL) and HL-7702 cells (10^6 cells/mL) were planted into 96-well microtiter plates in DMEM or RPMI 1640 with 10% fetal bovine serum (FBS). Plates were maintained at 37 °C in a 5% CO_2/95% air incubator for 24 h. Then the cells were incubated for 24 h at 37 °C in a 5% CO_2/95% air upon different concentrations probe of 0 μM to 100 μM respectively. MTT solution (5.0 mg/mL, PBS) was then added to each well. After 4 h, the remaining MTT solution was removed, and 200 μL of DMSO was added to each well, shaking 10 min to dissolve the formazan crystals at room temperature. Absorbance was measured at 490 nm in a TECAN infinite M200pro microplate reader.

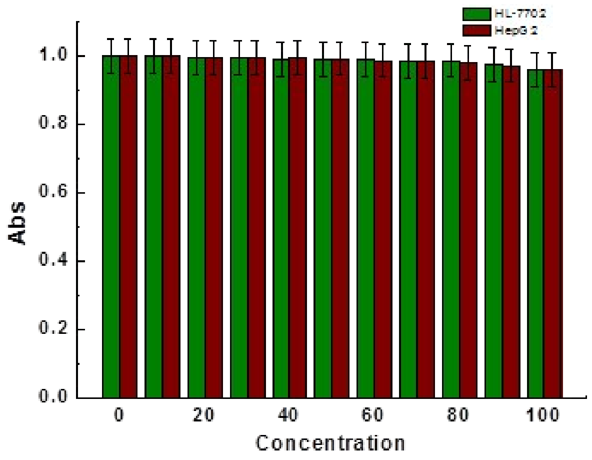

Fig. S7 MTT assay of HepG2 and HL-7702 cells with different concentrations of **CyO-Dise**. The experiment was repeated five times and the data were shown as mean (± s.d.).

9. Bright-field Images of Fig. 1

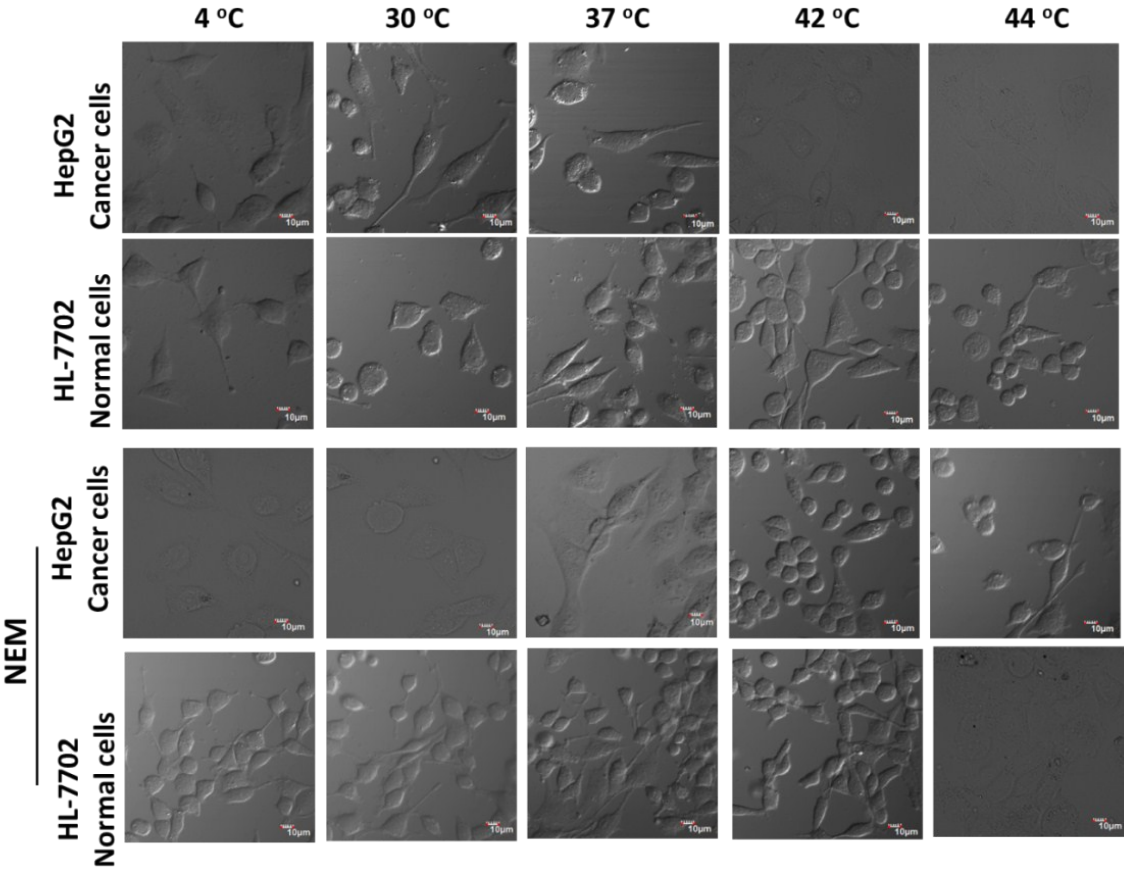

Fig. S8 Bright-field images of Fig. 1.

10. GSH concentrations in Fig. 1 Measured by CyO-Dise through Cell Imaging Experiments

Table S1 GSH concentrations in Fig. 1 measured by **CyO-Dise** through cell imaging experiments.

	Concentration (μM)				
Groups \ Temperature	4℃	30℃	37℃	42℃	44℃
HepG2	5.92	5.59	5.22	5.47	5.81
HL-7702	6.22	5.81	5.35	5.70	5.92
NEM-HepG2	0	0	0	0	0
NEM-HL-7702	0	0	0	0	0

11. GSH concentrations in Fig. 1 Measured by CyO-Dise through Flow Cytometry Analysis

Table S2 GSH concentrations in Fig. 1 measured by **CyO-Dise** through flow cytometry analysis.

Groups \ Temperature	Concentration (μM)				
	4℃	30℃	37℃	42℃	44℃
HepG2	6.21	5.81	5.35	5.70	5.92
HL-7702	6.46	6.02	5.70	5.92	6.11
NEM-HepG2	0	0	0	0	0
NEM-HL-7702	0	0	0	0	0

12. GSH concentrations in Fig. 1 measured by Total Glutathione Assay Kit

The GSH concentrations in Fig. 1 were further measured by Total Glutathione Assay Kit (Beyotime, China). The total GSH concentrations were shown in Table S3, which was consistent with our probe analysis results.

Table S3 GSH concentrations in Fig. 1 measured by Total Glutathione Assay Kit.

Groups \ Temperature	Concentration (μM)				
	4℃	30℃	37℃	42℃	44℃
HepG2	6.42	5.92	5.41	5.85	6.01
HL-7702	6.62	6.24	5.93	6.05	6.16
NEM-HepG2	0.81	0.78	0.8	0.8	0.7
NEM-HL-7702	0.88	0.79	0.88	0.83	0.75

13. Bright-field Images of Fig. 2

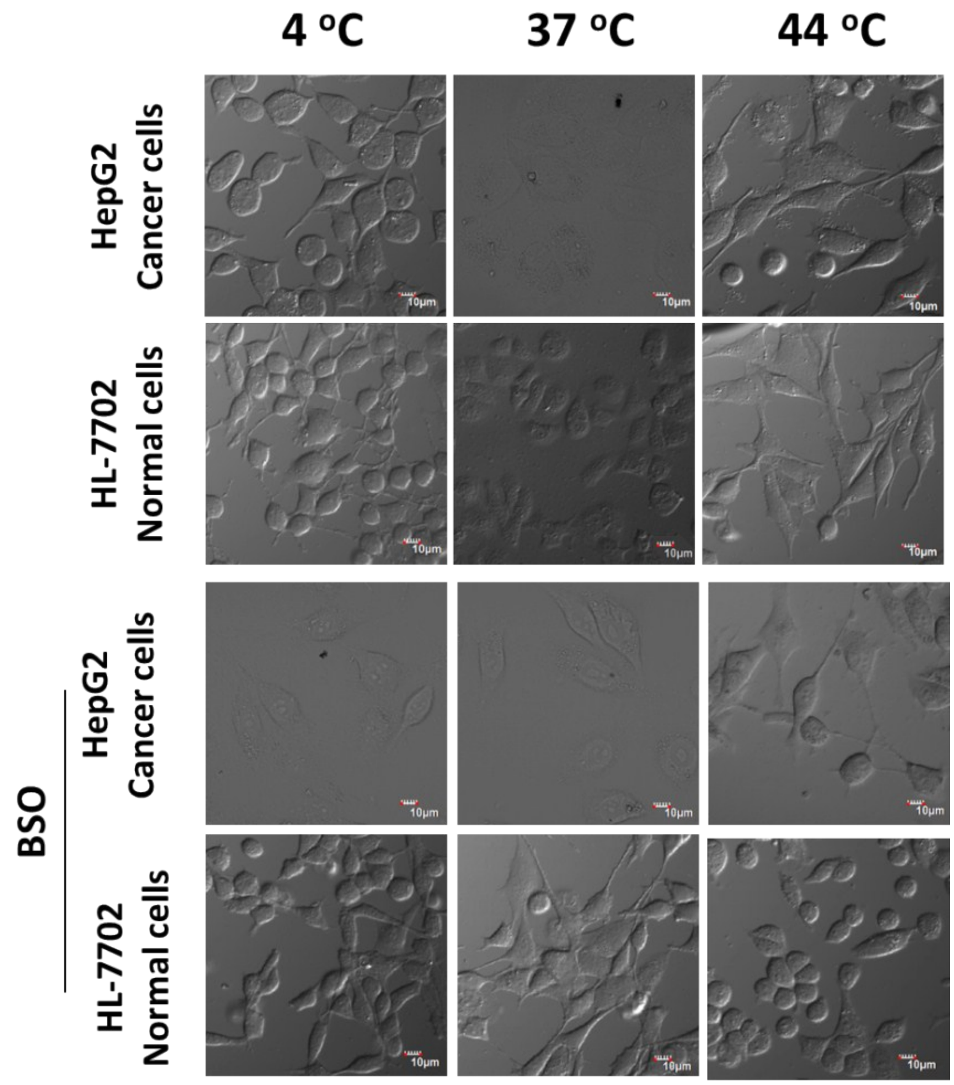

Fig. S9 Bright-field images of Fig. 2.

14. Plots of average ratio intensities CyO-Dise in Fig. 2a

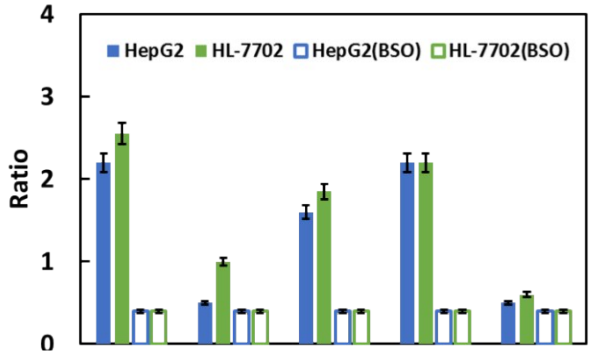

Fig. S10 Plots of average ratio intensities **CyO-Dise** in Fig. 2a.

15. Flow Cytometry Analysis of GSH Concentrations by CyO-Dise for Fig. 2

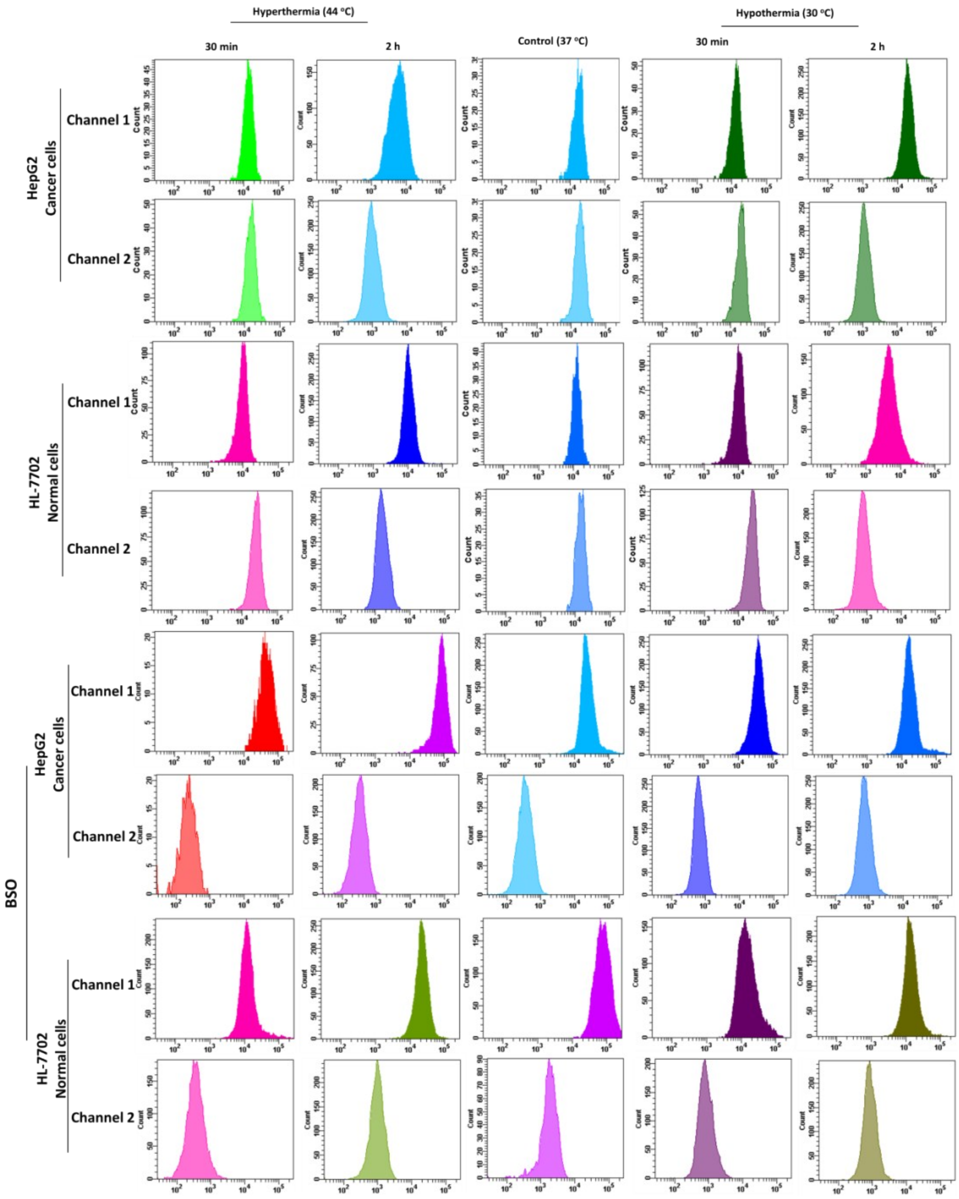

Fig. S11 Flow cytometry analysis of GSH concentrations by **CyO-Dise** for Fig. 2.

16. Flow Cytometry Analysis of Ca^{2+} Concentration by Fluo 4-AM for Fig. 2

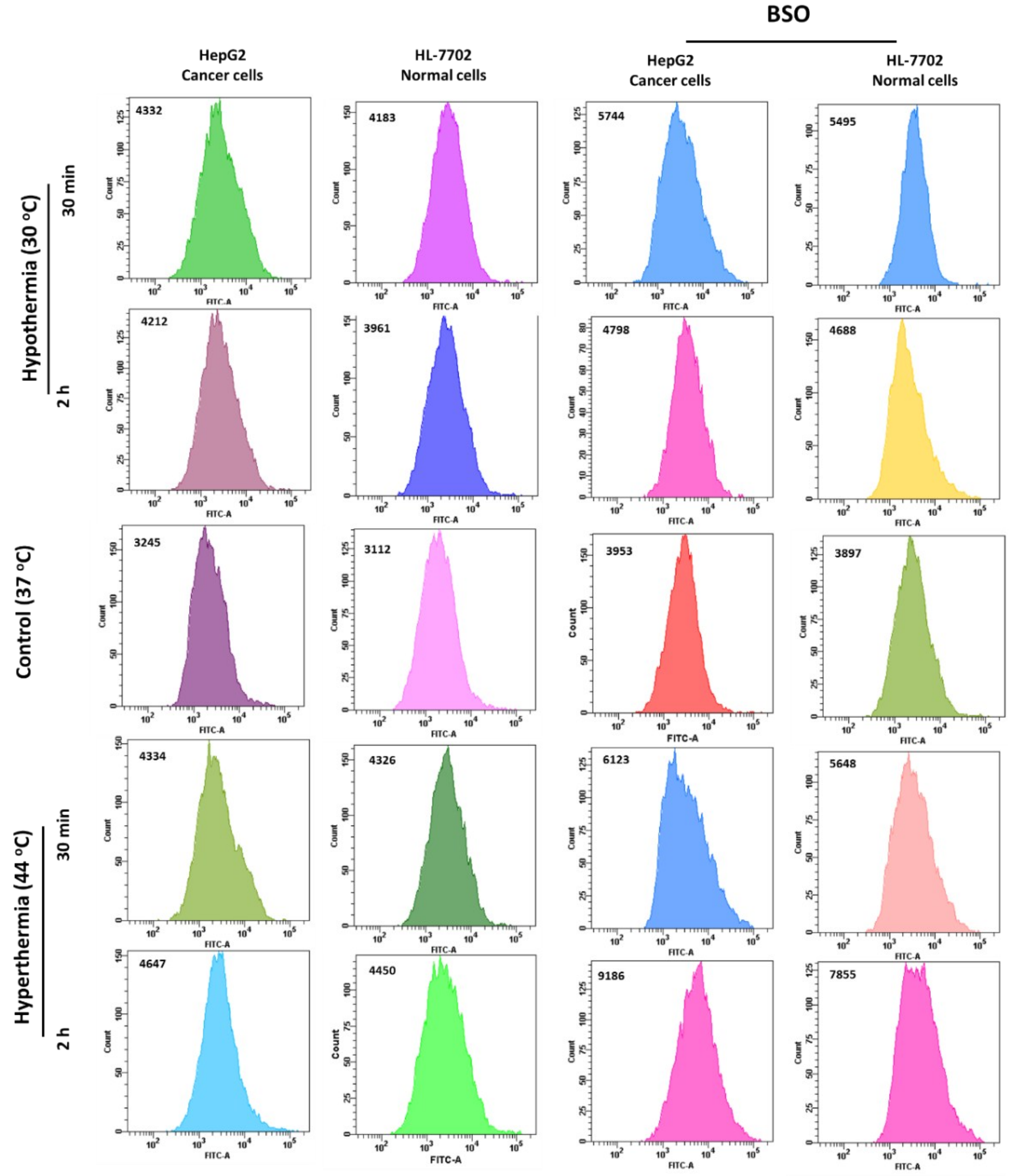

Fig. S12 Flow cytometry analysis of Ca^{2+} concentration by Fluo 4-AM for Fig. 2.

17. Transmission Electron Microscopy Observation of Cells in Fig. 2

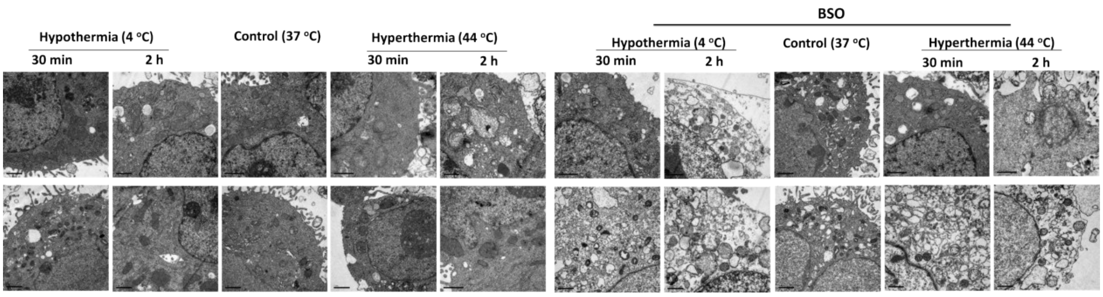

Fig. S13 Transmission electron microscopy observation of cells in Fig. 2 (Scale bar: 1 μm).

18. Evaluation of the anti-apoptosis effect of GSH upon exogenous cysteine under hypothermia and hyperthermia

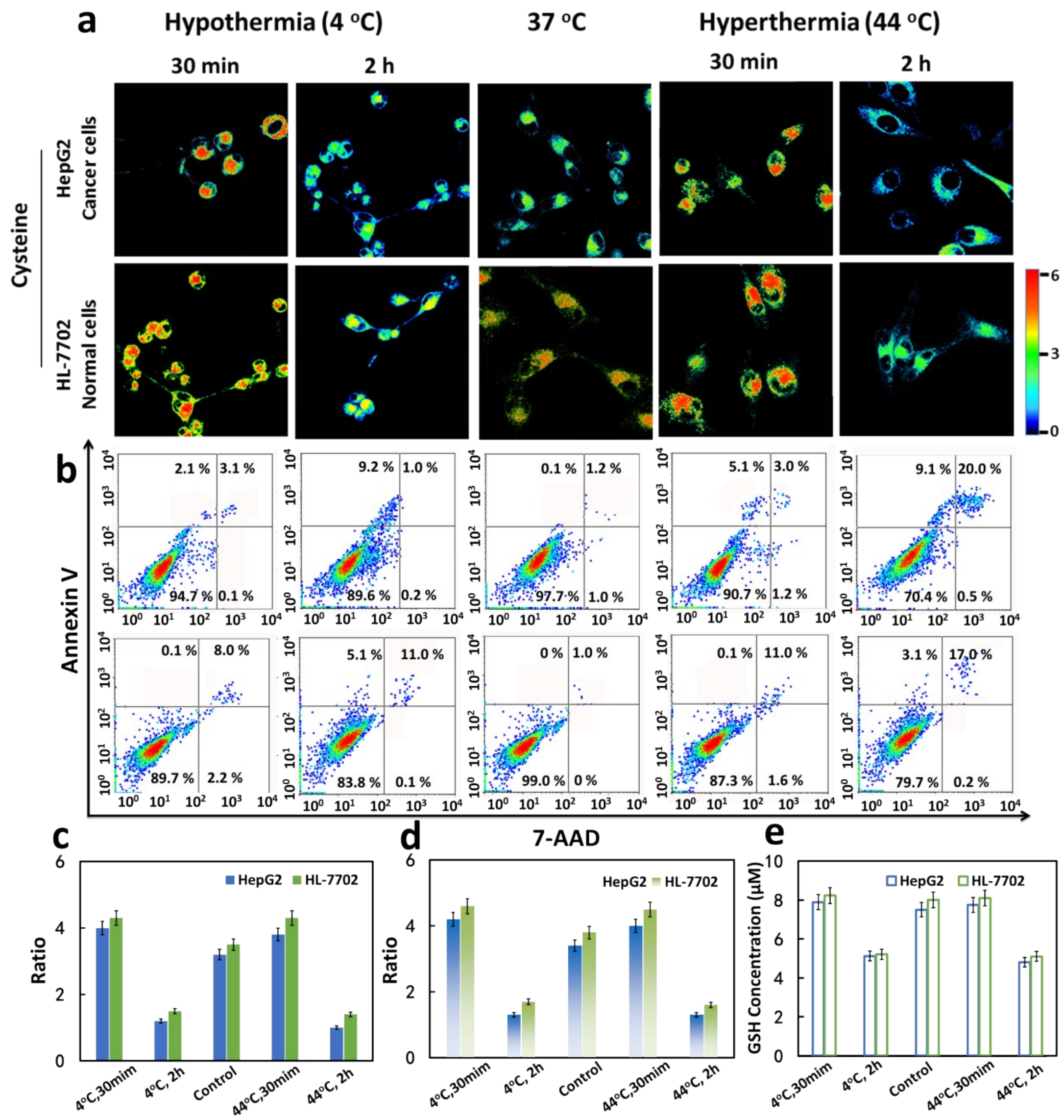

Fig. S14 Evaluation of the anti-apoptosis effect of GSH upon exogenous cysteine under hypothermia and hyperthermia. The cells were incubated with 100 μM cysteine for 30 min before the same treat as Fig. 3. a) Pseudo-color ratio images. b) Apoptosis analysis by Annexin V/7-AAD: viable cells (AnnexinV-/7-AAD-), early apoptosis (AnnexinV+/7-AAD-), late apoptosis (AnnexinV+/7-AAD+), necrosis (AnnexinV-/7-AAD+). c) Histograms of average ratio intensities in a). d) Flow cytometry analysis of GSH concentrations by **CyO-Dise**. e) GSH concentrations in Fig. S14a measured by Total Glutathione Assay Kit. The experiments were repeated three times (n = 7 per test) and the data were shown as mean (± s.d.).

Table S4 GSH concentrations in Fig. S14c measured by **CyO-Dise** through cell imaging experiments.

	Concentration (mM)				
Groups \ Temperature	4℃ 30 min	4℃ 2 min	37℃	44℃ 2 h	44℃ 2 h
HepG2	7.11	4.04	6.46	6.98	3.55
HL-7702	7.30	4.61	7.11	7.28	4.44

Table S5 GSH concentrations in Fig. S14d measured by **CyO-Dise** through flow cytometry analysis.

	Concentration (mM)				
Groups \ Temperature	4℃ 30 min	4℃ 2 min	37℃	44℃ 2 h	44℃ 2 h
HepG2	7.24	4.25	6.55	7.11	3.58
HL-7702	7.47	4.93	7.24	7.42	3.68

19. Histograms of Protein Expression in Fig. 2k

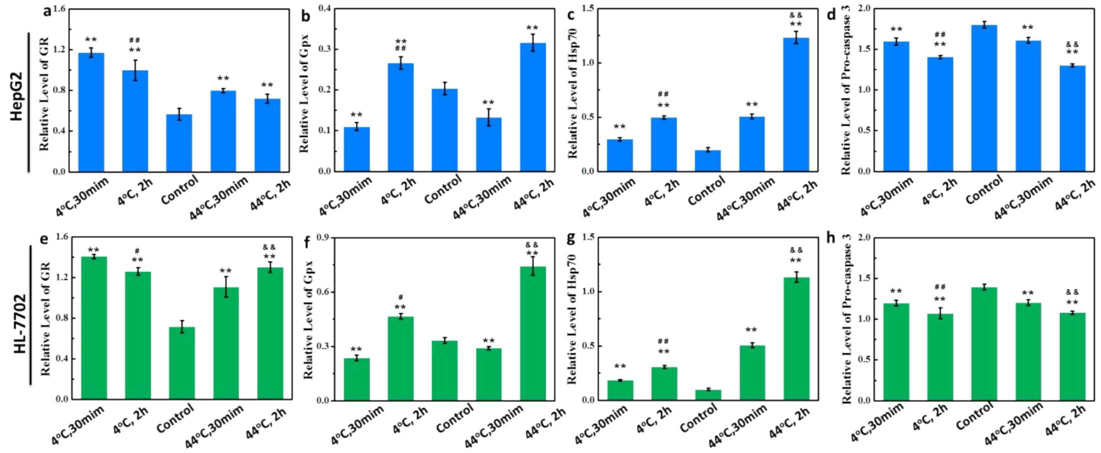

Fig. S15 A statistical analysis of protein expression in Fig. 2k. Upper row: the expression of GR (a), Gpx (b), Hsp70 (c) and Pro-caspase 3 (d) in HepG2 cells. Bottom row: the expression of GR (a), Gpx (b), Hsp70 (c) and Pro-caspase 3 (d) in HL-7702 cells. The experiments were repeated seven times and the data were shown as mean (± s.d.). Difference was analyzed by one-way ANOVA. ➤$P < 0.05$, ➤➤$P < 0.01$ v.s. control group. #$P < 0.05$, ##$P < 0.01$ on the 4 °C and 30 min group v.s. 4 °C and 2 h group. &$P < 0.05$, &&$P < 0.01$ on the 44 °C and 30 min group v.s. 44 °C and 2 h group.

20. Determination the IC_{50} of HepG2/DDP to DDP with MTT assay

HepG2/DDP cells (5 × 10^4 cells/mL) were seeded in 96-well microplates, at 100 μl/well and cultured overnight to allow cell adherence. Then, gradient concentrations of DDP (0, 20, 40, 60, 80, 100, 120, 140, 160, 180, 200, 220, 240, 260, 280, 300 μM) were added and the cells were further cultured for 72 h. Next, 50 μl MTT was added in accordance with the reagent instructions and cultured for 4 h. The optical density (OD) was measured at 490 nm wavelength with a TECAN infinite M200pro microplate reader. The inhibition rate of the drug on the cells was calculated as follows:

Using the DDP concentration as abscissa and the inhibition rate as ordinate, the inhibition curves were plotted

and fitted to obtain the half maximal inhibitory concentrations (IC_{50}).

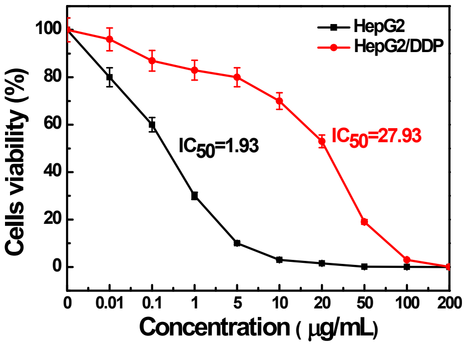

Fig. S16 IC_{50} value of HepG2 cells and HepG2/DDP cells. All assays were repeated at least seven times.

21. Bright-field Images of Fig. 3a

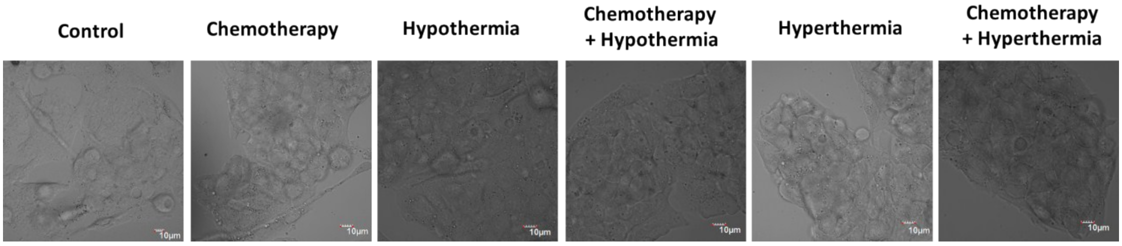

Fig. S17 Bright-field images of Fig. 3a.

22. Flow Cytometry Analysis of GSH Concentrations by CyO-Dise for Fig. 3

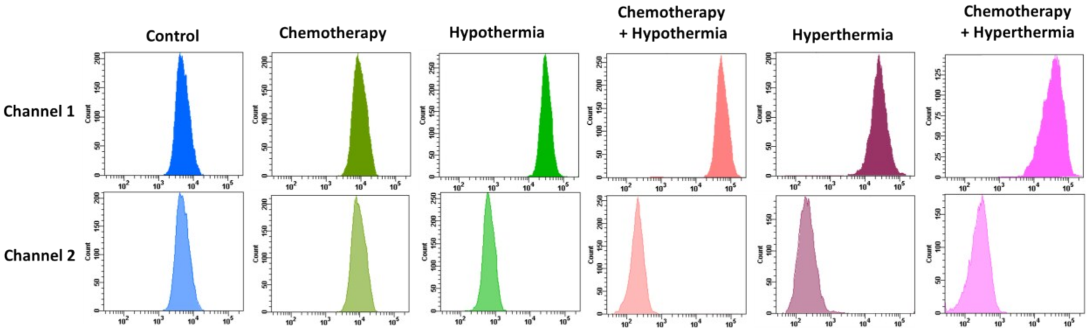

Fig. S18 Flow cytometry analysis of GSH concentrations by **CyO-Dise** for Fig. 3.

23. Flow Cytometry Analysis of Ca^{2+} Concentrations by Fluo 4-AM for Fig. 3

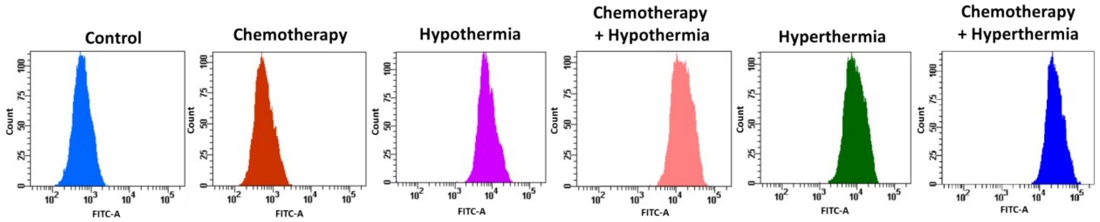

Fig. S19 Flow cytometry analysis of Ca^{2+} concentration by Fluo 4-AM for Fig. 3.

24. A Statistical Analysis Protein Expression in Fig. 3

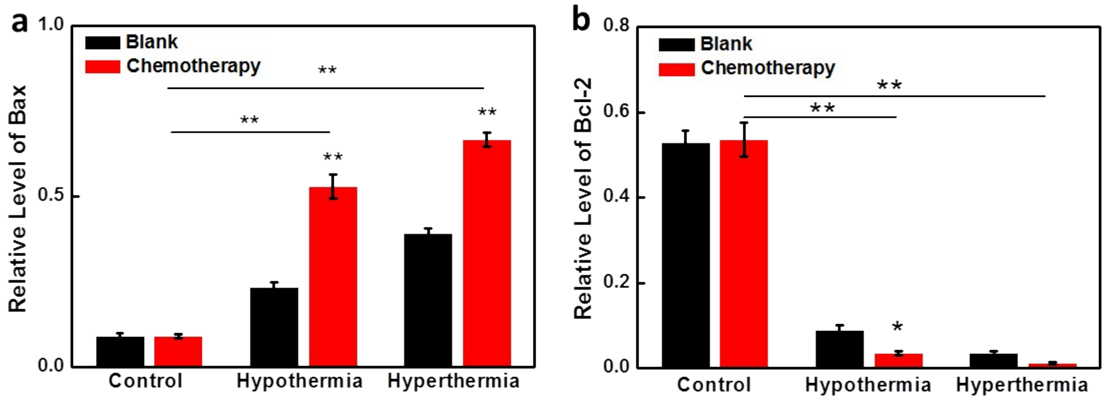

Fig. S20 A statistical analysis of protein expression in Fig. 3. (a) A statistical analysis of Bax protein expression. (b) A statistical analysis of Bcl-2 protein expression. The data were shown as mean (± s.d.) (n = 7). The differences between the data were analyzed by two-way ANOVA. ➤$P < 0.05$, ➤➤$P < 0.01$.

25. A Statistical Analysis of GSH Concentrations by CyO-Dise in Fig. 3a

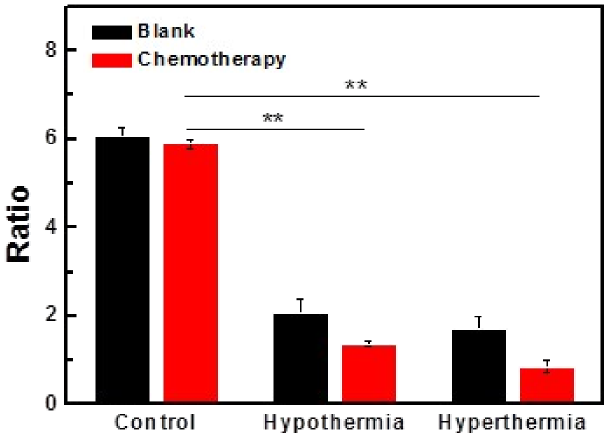

Fig. S21 A statistical analysis of GSH concentrations by **CyO-Dise** in Fig. 3a.

26. Evaluation of the anti-apoptosis effect of GSH in drug-resistant cells upon exogenous cysteine under hypothermia and hyperthermia

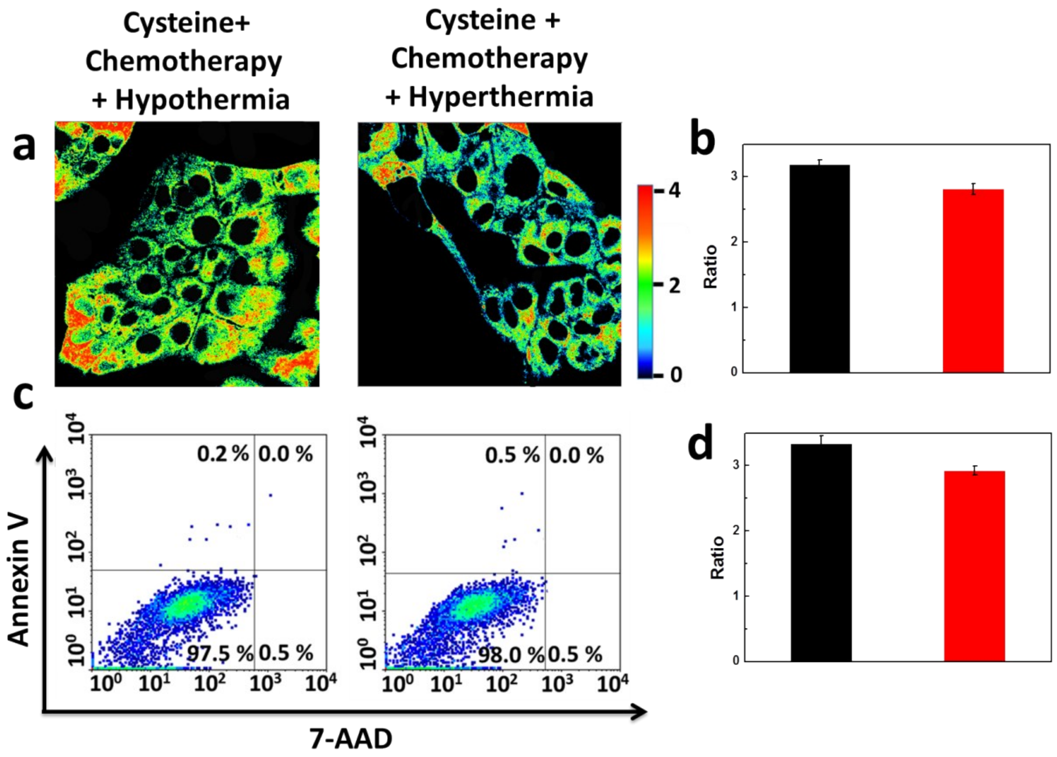

Fig. S22 Evaluation of the anti-apoptosis effect of GSH in drug-resistant cells upon exogenous cysteine under hypothermia and hyperthermia. The cells were incubated with 100 μM cysteine for 30 min before treated with Chemotherapy + Hypothermia/Hyperthermia. a) Pseudo-color ratio images. b) Apoptosis analysis by Annexin V/7-AAD: viable cells (AnnexinV-/7-AAD-), early apoptosis (AnnexinV+/7-AAD-), late apoptosis (AnnexinV+/7-AAD+), necrosis (AnnexinV-/7-AAD+). c) A statistical analysis of GSH concentrations by **CyO-Dise** in a). d) Flow cytometry analysis of GSH concentrations by **CyO-Dise**. e) GSH concentrations measured by Total Glutathione Assay Kit. The experiments were repeated three times (n = 7 per test) and the data were shown as mean (± s.d.).

Table S6 GSH concentrations in Fig. S14 measured through cell imaging, flow cytometry analysis, and Total Glutathione Assay Kit.

	Concentration (μM)	
**Groups**	**Cysteine + Chemotherapy + Hypothermia**	**Cysteine + Chemotherapy + Hyperthermia**
**Cell Imaging**	**6.54**	**6.22**
**Flow Cytometry Analysis**	**6.65**	**6.32**
**Total Glutathione Assay Kit**	**6.97**	**6.59**

27. Evaluation of GSH Effiency in HepG2/DDP Subcutaneous Tumor Xenografts upon Triple Therapeutics.

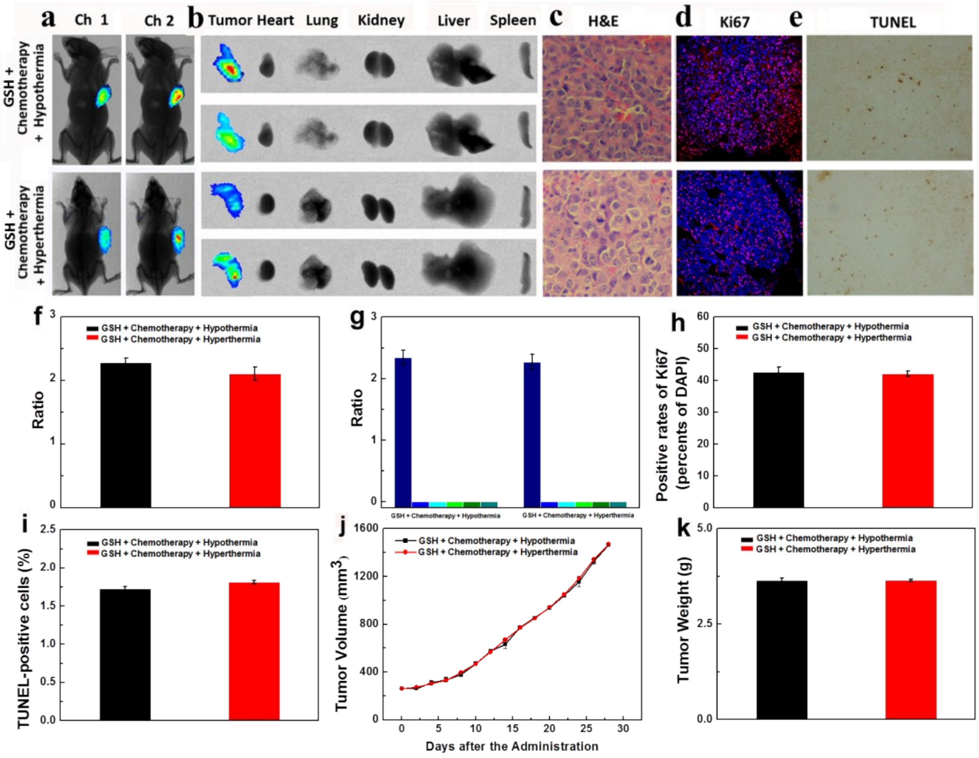

Fig. S23 Evaluation of GSH efficacy in HepG2/DDP subcutaneous tumor xenografts upon triple therapeutics. (a) *In vivo* imaging for 30 min after tail vein injection of a single-dose 0.2 ml of **CyO-Dise** (DMSO/saline 1:1/v/v) (n = 7 per group). (b) *Ex vivo* imaging of GSH in separated organs (lung, heart, liver, kidney, spleen) and tumors sacrificed from (a). (c) Representative slides of H&E-stained tumors sacrificed from (a). Magnification: × 400. (d) Immunofluorescence staining of proliferation markers Ki67 (red channel: $\lambda_{ex/em}$ = 635/670-770 nm) with anti-Ki67 mAb (Alexa Fluor 647 Conjugate) and nucleus (blue channel: $\lambda_{ex/em}$ = 405/410-490 nm) with DAPI of HepG2/DDP subcutaneous tumor xenografts sections. Scale bar: 50 μm. (e) TUNEL staining of HepG2/DDP subcutaneous tumor xenografts sections. Magnification: × 100. (f) Histograms of average ratio intensities in (a). (g) Histograms of average ratio analysis of corresponding organs in (b). (h) Histograms of the data derived from (d). The error bars shown in the figures represented the mean ± s.d. (i) Histograms of the data derived from (e). The error bars shown in the figures represented the mean ± s.d. (j) Tumor sizes. The calculation of the volume followed the formula: volume=length × width^2 × 0.5. (k) Tumors mean weights. The experiments were repeated three times (n = 7 per tset) and the data were shown as mean (± s.d.).

28. Bright-field Images of Fig. 4d，Fig. 5d, and Fig. S23d

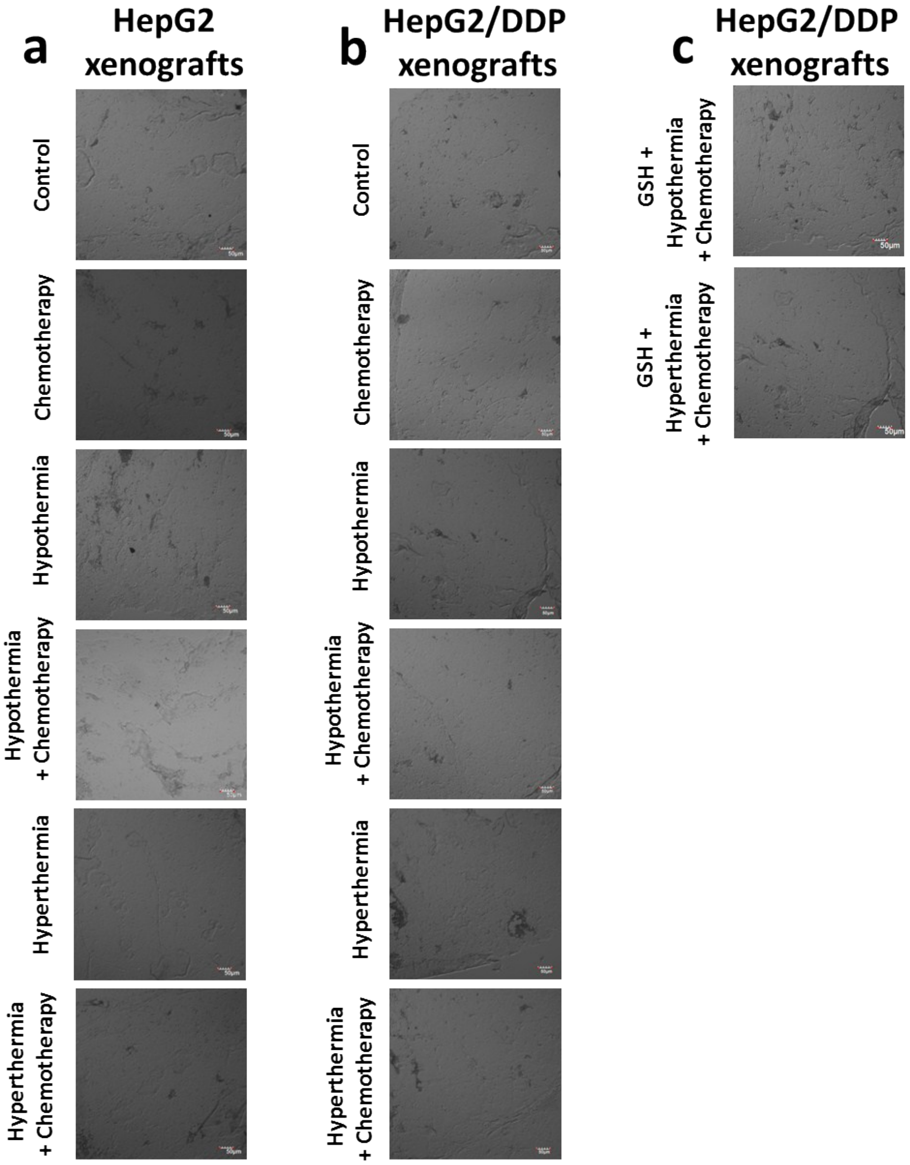

Fig. S24 Bright-field images of Fig. 4d (a), Fig. 5d (b) and Fig. S23d (c).

29. Body weight of Tumor-bearing Nude Mice in Fig. 4d，Fig. 5d, and Fig. S23d

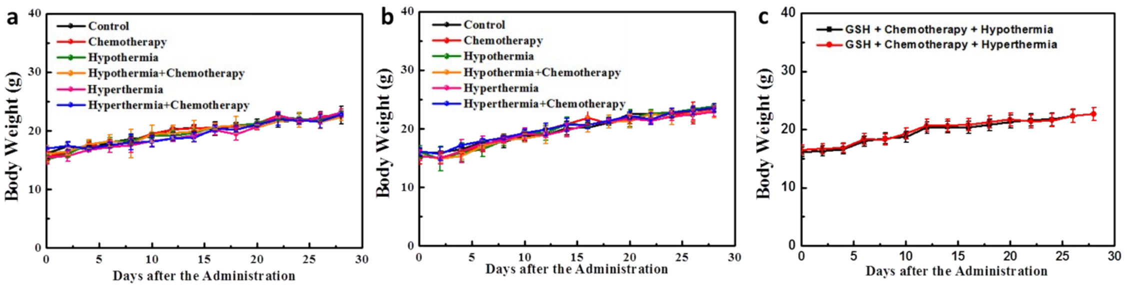

Fig. S25 Body weight of xenograft tumors. (a) HepG2 xenograft in Fig. 4. (b) HepG2/DDP xenograft in Fig. 5. (c) HepG2/DDP xenograft in Fig. S23. The error bars shown in the figures represented the mean ± s.d.

30. Fluorescent Recovery Assay

We performed the cell imaging experiments to check the fluorescence recovery. The cells were treated with 5 mM NEM for 30 min to consume all the GSH. And then 5 mM GSH monoethyl ester (GSH-MEE) were added for 2 h as exogenous GSH supplement.[11,12] Hypothermia (4 ^{o}C and 30 ^{o}C) and hyperthermia (42 ^{o}C and 44 ^{o}C) were carried out for 30 min, respectively. As shown in Fig. S26a, the ratio-fluorescent intensity signals recovered after the addition of GSH-MEE. The cell imaging experiments indicated that probe **CyO-Dise** could capture the fluorescence recovery from exogenous addition of GSH-MEE. And flow cytometry analysis was carried out to verify the above results (Fig. S26b). The flow cytometry analysis results were consisted with pseudo-color ratio images results. The average fluorescence intensities of **CyO-Dise** upon addition of GSH-MEE in Fig. 26a and the corresponding mean ratio intensities in Fig. 26b were further shown in Fig. 26c and Fig. 26d. After calculations using the regression equation in Fig. S1d, we obtained concentrations of GSH in HL-7702 and HepG2 cells, respectively (Table S7 and S8). All the results revealed that the probe **CyO-Dise** could be used as a facilitative tool to directly image GSH recovery in living cells.

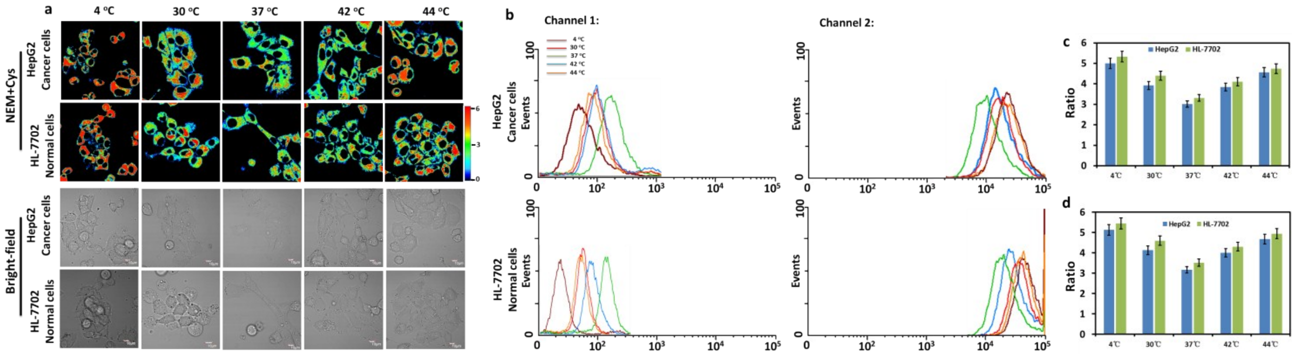

Fig. S26 Real-time GSH quantification with **CyO-Dise** (10 μM) in HepG2 and HL-7702 cells upon hypothermia (4 ^{o}C and 30 ^{o}C) and hyperthermia (42 ^{o}C and 44 ^{o}C) for 30 min. The cells were treated with 5 mM NEM for 30 min and then incubated with 5 mM GSH-MEE for 2 h. a) Pseudo-color ratio images. b) Flow cytometry analysis. c) Histograms of average ratio intensities in a). d) Corresponding mean ratio intensity in b). The data were shown as mean (± s.d.) (n = 7).

Table S7 GSH concentrations in Fig. S26a measured by **CyO-Dise** through cell imaging experiments.

	Concentration (mM)				
Groups \ Temperature	4℃	30℃	37℃	42℃	44℃
GSH-MEE-HepG2	7.68	7.06	6.39	7.02	7.45
GSH-MEE-HL-7702	7.85	7.35	6.63	7.18	7.54

Table S8 GSH concentrations in Fig. S26b measured by **CyO-Dise** through flow cytometry analysis.

	Concentration (mM)				
Groups \ Temperature	4℃	30℃	37℃	42℃	44℃
GSH-MEE-HepG2	7.74	7.19	6.52	7.11	7.51
GSH-MEE-HL-7702	7.90	7.47	6.78	7.30	7.65

31. HPLC Data for Quantification GSH Levels

To check the authenticity and accuracy of the quantification results, we further performed high-performance liquid chromatography (HPLC) experiments. 5,5'-dithio-bis-nitrobenzoic acid (DTNB) was introduced as precolumn derivatization reagent as shown in Scheme S4.

The cultured HepG2, HL-7702 cells and HepG2/DDP were treated as described and then washed with PBS buffer twice. After centrifugation and removal of supernatant liquid, the resulting cells were stored at -80°C for 5 min. After thawing to room temperature, the cells were lysed by hyperacoustic in 1 mL lysis buffer (PBS with 0.1% Triton X-100, pH 7.4) and the supernatant liquid was collected after centrifugation (10,000 × *g*, 10 min, 4 °C). The derivatization reaction was realized utilizing 100 µL of the cells supernatant, 500 µL of Tris–HCl buffer 0.5 M, pH 8.9 and 300 µL of DTNB 10 mM in of K_2HPO_4 0.5 M, pH 8.0. After 5 min in an ice bath, was acidified by addition of 100 µL H_3PO_4 7 M and centrifuged at 3000× *g* for 10 min.[13]Then, derivatized samples were filtered through a 0.22 µm membrane for HPLC analysis. The derivatized samples were separated on RP-HPLC with a ZORBAX SB-C18 column (4.6 × 150 mm, 5 µm). Mobile phase A consisted of 12% methanol (v/v), 100 mM KH_2PO_4, pH 3.8 at a flow rate of 0.8 mL/min. Mobile phase B consisted of 40% methanol(v/v), 100 mM KH_2PO_4, pH 3.8 at a flow rate of 0.8 mL/min.[14] GSH-DTNB was detected by UV absorbance at 330 nm. The column temperature was 37°C, and the injection volume was 20 µL.

GSH standards (2 mM, 4 mM, 6 mM, 8 mM and10 mM) were selected to perform HPLC analysis (Fig. S27a). Retention time of GSH-DTNB was 11.13 min. There existed a linear relationship between peak area and the concentrations of GSH (Fig. S27b). The regression equation was Area = 36. 5 [GSH] mM - 46.2, with r = 0.9937. HPLC analysis of GSH concentrations in Fig.1 and Fig.S26 were shown in Table S9. The GSH level changes in Fig.2 and Fig.S14 were demonstrated in Table S10. Table S11 revealed the accurate GSH concentration fluctuations in Fig.3 and Fig.S22.

Scheme S4. The derivative reaction between GSH and DTNB.

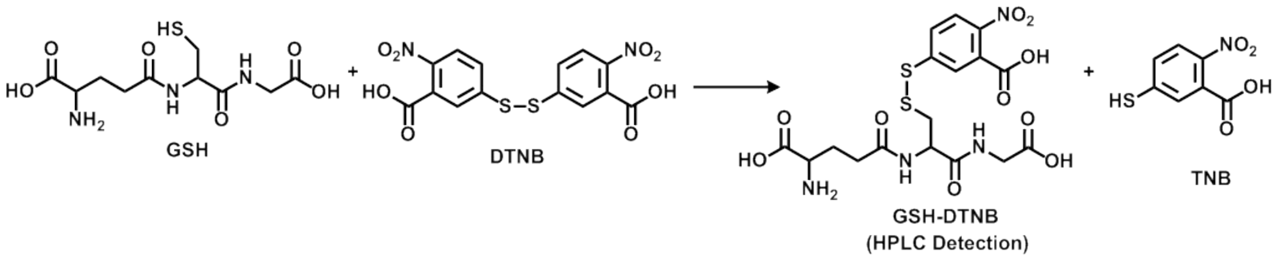

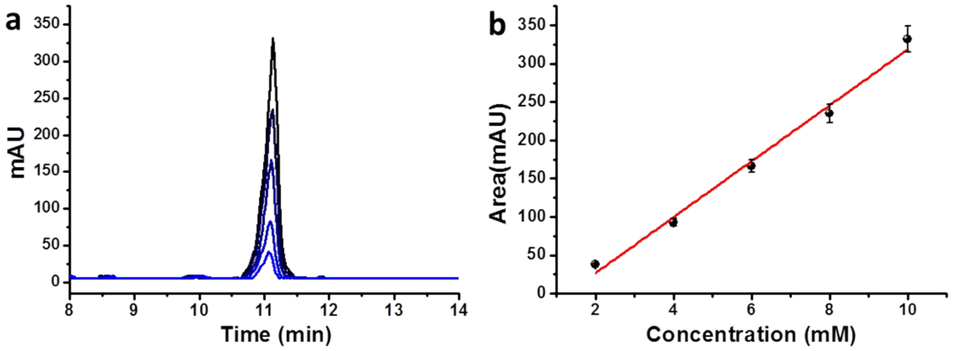

Fig. S27 a) HPLC analysis of GSH standards. b) Linear relationship between peak area and the concentrations of GSH. The data were shown as mean (± s.d.) (n = 7).

Table S9 HPLC analysis of GSH concentrations in Fig.1 and Fig.S26.

	Concentration (mM)				
Temperature / Groups	4℃	30℃	37℃	42℃	44℃
**HepG2**	**6.33 ± 0.12**	**5.91 ± 0.11**	**5.41 ± 0.13**	**5.78 ± 0.15**	**5.95 ± 0.12**
**HL-7702**	**6.55 ± 0.14**	**6.12 ± 0.19**	**5.88 ± 0.16**	**6.01 ± 0.21**	**6.13 ± 0.22**
**NEM-HepG2**	**0**	**0**	**0**	**0**	**0**
**NEM-HL-7702**	**0**	**0**	**0**	**0**	**0**
**GSH-MEE-HepG2**	**7.86 ± 0.15**	**7.33 ± 0.14**	**6.82 ± 0.15**	**7.13 ± 0.22**	**7.63 ± 0.33**
**GSH-MEE-HL-7702**	**7.99 ± 0.21**	**7.69 ± 0.11**	**6.93 ± 0.25**	**7.36 ± 0.28**	**7.82 ± 0.25**

Table S10 HPLC analysis of GSH concentrations in Fig.2 and Fig.S14.

	Concentration (mM)				
Temperature / Groups	4℃ 30 min	4℃ 2 min	37℃	44℃ 30 min	44℃ 2 h
**HepG2**	**6.33 ± 0.12**	**3.11 ± 0.11**	**5.41 ± 0.13**	**5.95 ± 0.12**	**2.29 ± 0.12**
**HL-7702**	**6.55 ± 0.14**	**3.88 ± 0.19**	**5.88 ± 0.16**	**6.13 ± 0.22**	**3.13 ± 0.22**
**BSO-HepG2**	**0**	**0**	**0**	**0**	**0**
**BSO-HL-7702**	**0**	**0**	**0**	**0**	**0**
**Cys-HepG2**	**7.35 ± 0.11**	**4.33 ± 0.14**	**6.62 ± 0.12**	**7.23 ± 0.12**	**3.63 ± 0.13**
**Cys-HL-7702**	**7.59 ± 0.18**	**5.19 ± 0.13**	**7.33 ± 0.22**	**7.46 ± 0.18**	**3.72 ± 0.12**

Table S11 HPLC analysis of GSH concentrations in Fig.3 and Fig.S22.

Groups	Concentration (mM)
**Control**	**8.33 ± 0.12**
**Chemotherapy**	**8.32 ± 0.20**
**Hypothermia**	**5.92 ± 0.28**
**Chemotherapy + Hypothermia**	**4.63 ± 0.23**
**Hyperthermia**	**5.19 ± 0.15**
**Chemotherapy + Hyperthermia**	**3.58 ± 0.19**
**Cysteine + Chemotherapy + Hypothermia**	**6.78 ± 0.13**
**Cysteine + Chemotherapy + Hyperthermia**	**6.42 ± 0.20**

32. References

1. S. Wu, T. Zhang, and J. Du, *Drug Des. Devel. Ther.,* 2016, **10**, 3471-3481.
2. F. Yu, M. Gao, M. Li and L. Chen, *Biomaterial*s, 2015, **63**, 93-101.
3. F. Amblard, J. H. Cho and R. F. Schinazi, *Chem. Rev.,* 2009, **109**, 4207-4220.
4. M. H. Lee, H. M. Jeon, J. H. Han, N. Park, C. Kang, J. L. Sessler and J. S. Kim, *J. Am. Chem. Soc.* 2014, **136**, 8430-8437.
5. X. Han, F. Yu, X. Song and L. Chen, *Chem. Sci.,* 2016, **7**, 5098-5107.
6. N. Couto, J. Wood and J. Barber, *Free Radic. Biol. Med.,* 2016, **95**, 27-42.
7. F. Yu, P. Li, P. Song, B. Wang, J. Zhao and K. Han, *Chem. Commun.,* 2012, **48**, 4980-4982.
8. Y. Qiao, K. Han, C. G. Zhan, *Biochemistry*, 2013, **52**, 6467−6479
9. M, J, Frisch, G. W. Trucks, et al. Gaussian 03, version C.02, Gaussian, Inc., Wallingford, CT, 2004.
10. D. A. Case, D. A. Plearman, J. W. Cadwell, T. E. I. I. I. Cheatham, J. Wang, W. Ross, C. L. Simmerling , T. A. Darden, K. M. Merz, AMBER7, 2002, San FranciscoUniversity of California.
11. J. Yin, Y. Kwon, D. Kim, D. Lee, G. Kim, Y. Hu, J. H. Ryu, J. Yoon, *Nat. Protoc.,* 2015, **10**, 1742-1754.
12. D. Lee, G. Kim, J. Yin, J. Yoon, *Chem. Commun.*, 2015, **51**, 6518-6520.
13. S. C. Garcia, K. Schott, M. Charão, A. Moro, R. Bulcão, D. Grotto, J. Valentini, D. Bohrer, S. Cardoso, V. Pomblum, *Biomed. Chromatogr.,* 2008, **22**, 460-468.
14. A. E. Katrusiak, P. G. Paterson, H. Kamencic, A. Shoker, A.W. Lyon, *J. Chromatogr. B*, 2001, **758**, 207-212.
